# Using Smart Displays to Implement an eHealth System for Older Adults With Multiple Chronic Conditions: Protocol for a Randomized Controlled Trial

**DOI:** 10.2196/37522

**Published:** 2022-05-05

**Authors:** David H Gustafson, Marie-Louise Mares, Darcie C Johnston, Gina Landucci, Klaren Pe-Romashko, Olivia J Vjorn, Yaxin Hu, David H Gustafson, Adam Maus, Jane E Mahoney, Bilge Mutlu

**Affiliations:** 1 Center for Health Enhancement Systems Studies University of Wisconsin–Madison Madison, WI United States; 2 Department of Industrial and Systems Engineering University of Wisconsin–Madison Madison, WI United States; 3 Department of Communication Arts University of Wisconsin–Madison Madison, WI United States; 4 Department of Computer Sciences University of Wisconsin–Madison Madison, WI United States; 5 Department of Medicine School of Medicine and Public Health University of Wisconsin Madison, WI United States

**Keywords:** eHealth, aged, geriatrics, multiple chronic conditions, chronic pain, smart displays, smart speakers, quality of life, primary care, health expenditures, mobile phone

## Abstract

**Background:**

Voice-controlled smart speakers and displays have a unique but unproven potential for delivering eHealth interventions. Many laptop- and smartphone-based interventions have been shown to improve multiple outcomes, but voice-controlled platforms have not been tested in large-scale rigorous trials. Older adults with multiple chronic health conditions, who need tools to help with their daily management, may be especially good candidates for interventions on voice-controlled devices because these patients often have physical limitations, such as tremors or vision problems, that make the use of laptops and smartphones challenging.

**Objective:**

The aim of this study is to assess whether participants using an evidence-based intervention (ElderTree) on a smart display will experience decreased pain interference and improved quality of life and related measures in comparison with participants using ElderTree on a laptop and control participants who are given no device or access to ElderTree.

**Methods:**

A total of 291 adults aged ≥60 years with chronic pain and ≥3 additional chronic conditions will be recruited from primary care clinics and community organizations and randomized 1:1:1 to ElderTree access on a smart display along with their usual care, ElderTree access on a touch screen laptop along with usual care, or usual care alone. All patients will be followed for 8 months. The primary outcomes are differences between groups in measures of pain interference and psychosocial quality of life. The secondary outcomes are between-group differences in system use at 8 months, physical quality of life, pain intensity, hospital readmissions, communication with medical providers, health distress, well-being, loneliness, and irritability. We will also examine mediators and moderators of the effects of ElderTree on both platforms. At baseline, 4 months, and 8 months, patients will complete written surveys comprising validated scales selected for good psychometric properties with similar populations. ElderTree use data will be collected continuously in system logs. We will use linear mixed-effects models to evaluate outcomes over time, with treatment condition and time acting as between-participant factors. Separate analyses will be conducted for each outcome.

**Results:**

Recruitment began in August 2021 and will run through April 2023. The intervention period will end in December 2023. The findings will be disseminated via peer-reviewed publications.

**Conclusions:**

To our knowledge, this is the first study with a large sample and long time frame to examine whether a voice-controlled smart device can perform as well as or better than a laptop in implementing a health intervention for older patients with multiple chronic health conditions. As patients with multiple conditions are such a large cohort, the implications for cost as well as patient well-being are significant. Making the best use of current and developing technologies is a critical part of this effort.

**Trial Registration:**

ClinicalTrials.gov NCT04798196; https://clinicaltrials.gov/ct2/show/NCT04798196

**International Registered Report Identifier (IRRID):**

PRR1-10.2196/37522

## Introduction

### Background

The central question of this project concerns the effectiveness of different media platforms in delivering an eHealth intervention for older adults with multiple chronic conditions (MCCs), including pain. Thus far, eHealth interventions for chronic conditions have generally been delivered through computer websites or smartphone apps. It is unknown whether such interventions could be even more effective for older adults if delivered on emerging smart speakers and display platforms.

Smart speakers, such as Amazon Echo, are voice-controlled devices connected to the internet. Users talk to the device and listen to the results rather than typing and reading. Smart displays, such as Google Nest Hub, offer the same voice interactivity but add a screen to allow for viewing of videos, visual navigation of menu options, and touch selection of options. Both smart speakers and smart displays are increasingly designed to move beyond simple voice commands and responses to offer more conversational interactions via chatbots.

Various authors have speculated about the potential of these devices to transform self-management and treatment of chronic diseases such as diabetes [1-3]. For older adults with ongoing pain, limited mobility, limited vision, or hand tremors, interacting via voice may enhance accessibility. In theory, users could ask questions and have answers immediately spoken back to them. They could hear and respond to messages, request audio services such as guided meditation, record health data, and more just by talking or simple taps on the screen. At the same time, the chatbot interactivity could potentially enhance feelings of relationship and support offered by the device and by the specific eHealth intervention on the device. When asked to predict the near future of voice-activated devices in health care, a Delphi panel of 35 physicians, academics, and information technology specialists predicted that such devices would show notable gains in acceptance and use, particularly among older patients, because of their simplicity of use [4].

Indeed, media reports indicate increasing use of speakers among older adults and people with vision loss, tremors, lack of mobility, and dementia [5-9]. Thus far, a handful of studies, primarily conducted with samples of ≤20, suggest that older adults given smart devices generally report liking them, using them mostly for music and simple information requests such as the weather [10-15]. In a study of 12 older adults given a smart display for 16 weeks, the participants reported finding the device easier to use than a computer [12]. However, they also reported forgetting how to wake up the device, control the volume, or turn it off when it was activated unexpectedly, and device log data showed that one-third of attempted interactions were unsuccessful because the device did not understand the request. However, over time, users showed an increasing ability to rephrase unsuccessful requests, and transcriptions of interactions suggested companionable exchanges between the user and the device.

Thus far, only a couple of pilot studies have focused on health-related uses of smart speakers. In one, 58 patients with chronic conditions were given an Amazon Alexa device for 2 months, and a number of them reported using it to remind them to take their medications and to record when they had done so [16]. In a very similar study, 44 adults aged 50 to 90 years with chronic conditions were given Amazon’s Echo Show for 2 months [17]. They too reported using the device for medication and appointment reminders, with additional anecdotes of using it to find recipes tailored to dietary requirements, medication information, exercise videos, guided meditation, or soothing sounds to help with falling asleep. An unspecified subset of users felt that their health and quality of life had improved over the 2 months as a result of the device. Neither study involved a comparison group, reported on actual log data rather than self-reports of use, or systematically assessed health outcomes.

Given these promising small-scale findings, rigorous research seems warranted to examine whether smart displays can facilitate the self-management of chronic conditions. Chronic conditions are prevalent among older adults. Approximately two-thirds of Medicare beneficiaries have ≥3 ongoing conditions such as diabetes or arthritis, and nearly one-fourth have ≥5 [18,19]. The complexity of patients’ comorbidities makes them challenging to serve well in primary care, where time pressures and patient loads necessitate a focus on medication and laboratory results rather than well-being and skills for self-management, treatment adherence, and health tracking [20]. Patients with MCCs are also the most expensive, accounting for 90% of Medicare spending [18]. Most important for the individual patient, having multiple conditions is associated with reductions in physical and psychosocial quality of life [21-23] and increased risk of chronic pain [24].

Chronic pain, generally defined as pain lasting ≥3 months, is reciprocally intertwined with a number of variables [25] such as anxiety and depression [26,27], sleep [28], and daily functioning [29]. Chronic pain also intersects with issues of social justice; a recent review noted that those with lower socioeconomic status are more likely to report chronic pain, more severe pain, and more pain-related disability [24]. Various meta-analyses have indicated ongoing racial disparities, such that African Americans and other patients of color tend to be undertreated for pain [30,31]. Increased self-management of chronic pain is a national objective established by the US Department of Health and Human Services in *Healthy People 2030* for the next decade [32], and there is evidence that internet-based interventions can improve pain-related outcomes [33-35]. However, no research thus far has examined self-management interventions for chronic pain within the context of MCCs. Furthermore, there is no research on the relative effectiveness of different platforms for delivering such an intervention for this complex patient population.

### Need for a Trial

Large-scale, long-term trials of the effectiveness of delivery platforms may help advance the objective outlined in *Healthy People 2030*. This paper reports on the study design and methods of a randomized controlled trial of the effectiveness of an evidence-based eHealth intervention for older adults with MCCs, including pain, when delivered on a smart display versus a laptop platform.

The system, ElderTree, was developed by our Agency for Healthcare Research and Quality Center of Excellence in Active Aging to improve quality of life and socioemotional outcomes among older adults and was first tested in a randomized controlled trial involving 390 older adults who were followed for 12 months. In that intention-to-treat trial, patients in the ElderTree group who had had ≥3 primary care visits in the 6 months before baseline showed significantly better results on measures of mental quality of life, social support, and depression compared with control patients who had received a laptop but no ElderTree access [36].

Given that primary care use is relatively high among patients with MCCs, the results suggest that ElderTree may most benefit such patients and may be most effective if integrated into primary care. A subsequent randomized controlled trial, funded by the National Heart, Lung and Blood Institute, is currently testing ElderTree among patients with MCCs, with an added report to primary care clinicians documenting changes in health status. That ongoing trial is testing whether patients assigned to a laptop-based ElderTree versus an attention control will have better quality of life, fewer symptoms, and better condition-specific laboratory scores [37].

The trial described in this protocol is designed to test whether the delivery platform of the intervention affects its effectiveness. Specifically, the study aims to assess whether participants using ElderTree on a smart display will experience decreased pain interference and improved quality of life and related measures in comparison with participants using ElderTree on a laptop and control participants given no device or access to ElderTree.

## Methods

### Trial Design

The trial has a randomized controlled design with 3 groups with 1:1:1 allocation. Participants will be randomized to receive (1) a smart display plus internet access plus ElderTree along with their usual care, (2) a touch screen laptop plus internet access plus ElderTree along with their usual care, or (3) usual care alone. All participants will be followed for 8 months.

### Sample Size and Study Setting

A total of 291 older adults will be recruited from the University of Wisconsin–Madison Department of Family Medicine and General Internal Medicine system (UW Health), Access Community Health Centers, and community organizations in the Madison and Beloit, Wisconsin, areas.

### Intervention Groups

#### Control

Participants in the control arm will continue with their usual care and receive no device or intervention from the study.

#### ElderTree on a Smart Display Platform

In addition to continuing with their usual care, participants in the ElderTree on a smart display platform (ET-SD) arm will receive access to ElderTree for 8 months plus a Google Nest Hub Max smart device and internet access. The device consists of a voice-activated smart speaker and a 10-inch visual display that is optionally touch-activated.

#### ElderTree on a Laptop Platform

In addition to continuing their usual care, participants in the ElderTree on a laptop platform (ET-LT) arm will receive access to ElderTree for 8 months plus a touch screen laptop computer and internet access.

### Eligibility Criteria

Eligible participants will (1) be aged ≥60 years and (2) have chronic pain as indicated by having received a chronic pain diagnosis or reporting pain on some or most days that has lasted for ≥12 weeks (duration); reporting pain in the last 3 months on some, most, or all days (frequency); and reporting a pain intensity of ≥3 on a scale of 0 (no pain) to 10 (worst imaginable pain) in the last 7 days. They will also (3) have at least 3 of the most prevalent chronic conditions among older adults as reported by the Centers for Medicare and Medicaid Services [18]: chronic obstructive pulmonary disease, asthma, diabetes, hyperlipidemia, hypertension, ischemic heart disease, atrial fibrillation, heart failure, stroke, cancer, chronic kidney disease, depression, osteoporosis, and arthritis; we have modified the list by adding obesity (BMI≥30) and dizziness, falls, and loss of vestibular function. In addition, patients (4) will allow reports to their primary care provider about their health tracking and (5) should have no plans to leave during the study period. Patients are not eligible if they require an interpreter or if they have a medical diagnosis of Alzheimer disease, dementia, schizophrenia or other psychotic disorder, autism spectrum disorder, known terminal illness with <6 months to live, or an acute medical problem requiring immediate hospitalization**.**

### Recruitment

For UW Health recruitment, the university’s Clinical and Health Informatics Institute will use clinic records to identify patients meeting the aforementioned eligibility criteria and send their name, address, birthdate, age, UW Health clinic location, and primary care physician to the university’s Office of Clinical Trials via REDCap (Research Electronic Data Capture; Vanderbilt University). Potential participants will receive an opt-in letter describing the study and consent form from the Office of Clinical Trials plus a stamped return letter inviting contact from the study team.

We will supplement recruitment at UW Health with community efforts to increase the racial diversity of our patient population. The selection of and engagement with community organizations will be led by a senior advisor on our team with extensive experience in local health campaigns and deep roots in the local African American health care and patient communities. Through collaborations with the African American Health Network, Rebalanced-Life Wellness Association, African American Opioid Coalition, and the Community-Academic Aging Research Network, we will reach out to Black churches, community centers, fraternity and sorority health service programs, and other organizations as selected by our advisor in the Madison and Beloit areas of Wisconsin. We will distribute a recruitment flyer and consent form with a return card or opt-in and conduct video chat and in-person community sessions when possible to introduce the study and invite participation.

Through Access Community Health Centers, we expect to reach a larger number of African Americans and other participants of color as well as expand our reach to underserved patients. We will work directly with Access Centers staff to disseminate the recruitment flyer, consent form, and return card or opt-in to potential patients.

Regardless of the recruitment method, when a return card or opt-in is received, study staff will call and assess eligibility; provide a study overview that includes potential benefits and risks, study procedures, and compensation; thoroughly walk through informed consent; and address questions. The baseline survey will be mailed. Patients will be given adequate time to decide whether to participate and, in some cases, a follow-up phone call will be scheduled after the patient has had time to review the survey and consent form.

Once a patient verbally confirms that they want to participate, a home visit will be scheduled to collect the baseline survey, randomize, set up the equipment, and train. All study staff who conduct home visits will be fully vaccinated against COVID-19, wear face masks, and socially distance as much as possible while in the patient’s home. If no visit is desired, the equipment will be shipped once the study staff receives the completed baseline survey via mail, and technology setup and training will be conducted by phone. The baseline survey should take 20 to 30 minutes. The measures will be the same in all 3 arms to avoid differential dropout. We will document those who choose not to participate and why following the CONSORT (Consolidated Standards of Reporting Trials) guidelines.

### Randomization

**Figure 1:**
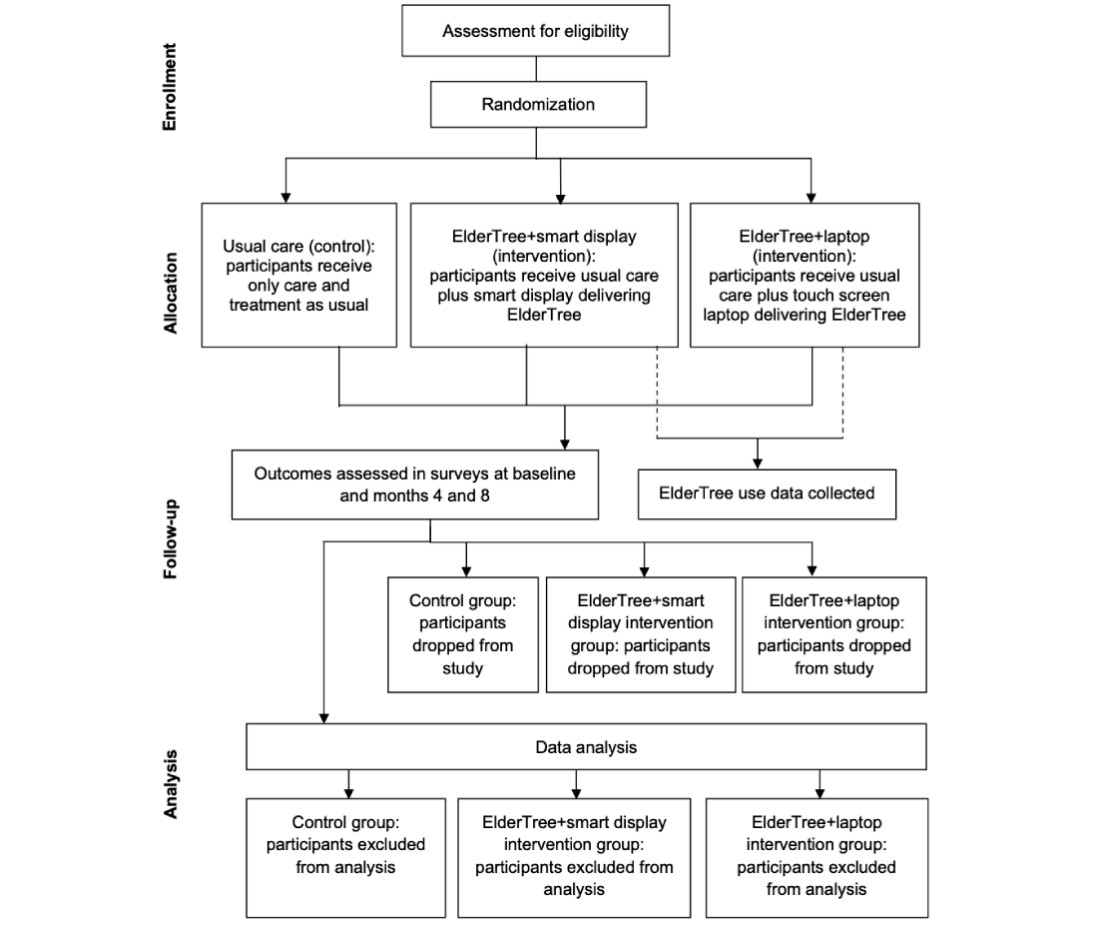
CONSORT (Consolidated Standards of Reporting Trials) flow diagram.

Once informed consent and the baseline survey have been obtained from patients, the project manager will use a computer-generated allocation sequence to randomize on a 1:1:1 ratio to the ET-SD, ET-LT, or control group stratified by sex (male or female), site (UW Health, Access Centers, or the community), and number of chronic conditions (≤5 or ≥6). A research staff member will then conduct equipment setup and training for patients based on group assignment, which is provided in a sealed opaque envelope. Obviously, once an assignment is made and a device has been set up, participants cannot be blinded to their condition. To set up participants on their assigned system (ET-SD or ET-LT), the researcher conducting the training cannot be blinded to the condition after assignment. Figure 1 shows the flow of participants during the trial.

### Timeline

Textbox 1 shows the timeline by year of the study, with year 1 beginning in August 2020 and year 5 ending in July 2025.

**Timeline of project activities.**

**Timeline and activities**

- Year 1, months 1 to 9: pilot-test smart display and complete development, develop content plan for laptop and smart display, prepare and finalize study and data collection materials
- Year 1, months 6 to 9: data quality monitor plan
- Year 1, months 7 to 9: receive institutional review board approvals, train research staff
- Year 1, month 7, to year 4, month 5: create and refresh content
- Year 2, month 1, to year 3, month 9: recruit, pretest, and randomize patients
- Year 2, month 1, to year 4, month 5: collect quantitative and qualitative data
- Year 2, month 9, to year 4, month 12: clean and prepare data
- Year 2, month 9, to year 5, month 12: analyze results
- Year 3, month 12, to year 5, month 12: publish

### Intervention

#### Background

For more than 30 years, our center has been developing and testing an evolving suite of eHealth systems collectively known as the Comprehensive Health Enhancement Support System (CHESS). ElderTree is one of these systems. All CHESS systems are built on principles of continuing care and self-management: long duration [38]; assertive outreach [39]; tracking [40]; prompts [41]; action planning [42]; problem solving and self-tailoring [43]; peer, family, and clinical support [44]; and care coordination [45]. In randomized trials, CHESS systems have significantly improved asthma control [46]; quality of life and cost of care in patients with HIV [47]; quality of life and self-efficacy in patients with breast cancer, including older women [48], compared with control [49] and internet [50] groups; risky drinking [51]; and caregiver burden, symptom distress, and median length of survival in patients with lung cancer [52].

#### System Overview

ElderTree is designed for older adults and with their input to offer tools, motivation, and social support for helping patients manage chronic pain and other chronic conditions. As reported in previous and ongoing studies of ElderTree [36,37], the laptop system is a members-only website free of advertisements with design features based on older users’ feedback as well as best practices for older populations, such as larger fonts, fewer options, and uncluttered screens for better comprehension, navigation, and usability [53]. The newly developed ET-SD version adheres to the same principles of effective interventions and system design (eg, uncluttered screens and fewer options) for older adults. Both systems substantially replicate ElderTree as described in our ongoing study comparing ET-LT with an attention control [37], with modifications and enhancements to focus on chronic pain and align with the technical capabilities of the smart platform.

#### Theoretical Foundation

ElderTree and other CHESS systems are consistent with self-determination theory, which asserts that satisfying 3 basic psychological needs contributes to adaptive functioning: competence (feeling effective, not overwhelmed), social relatedness (feeling connected to others, not isolated), and intrinsic motivation (feeling autonomous, not coerced) [54].

#### Interface and Features

The key features of the site and how they align with self-determination theory are described in Table 1.

**Table 1:** Key features and theoretical basis of the ElderTree eHealth intervention.

Feature or function	Description	Self-determination theory construct
Living Well with Chronic Pain course	Eight 20-minute learning modules with the latest findings on pain and evidence-based coping strategies	Health-related coping competence; motivation
Health library and wellness activities	Relaxation, meditation, physical exercise (eg, chair yoga), activity, and informational videos in relevant topic areas	Health-related coping competence; motivation
Weekly survey and clinician report	Tracking of self-reported general health measures (eg, sleep, mood, and exercise), graphed over time available for patients and their primary care clinicians	Health-related coping competence; motivation
Discussion groups (all)	Monitored support and chat forums [55,56]	Social relatedness and support
Chronic pain discussion group	Participants’ shared tips, experiences, and resources for managing chronic pain [57]	Health-related coping competence; social relatedness and support; motivation
Journal	Interactive function with prompts based on positive psychology principles [58]	Health-related coping competence; motivation
Thought of the day	Daily motivational and inspirational prompts	Motivation
Comment functionality	Posting function to promote engagement and relationship building on all information and activity pages	Social relatedness and support
Notifications and reminders	Custom reminders to take the weekly survey of health indicators; email and system notifications of responses to the user’s posts	Health-related coping competence

### Outcomes and Variables

#### Primary Outcomes

**Figure 2:**
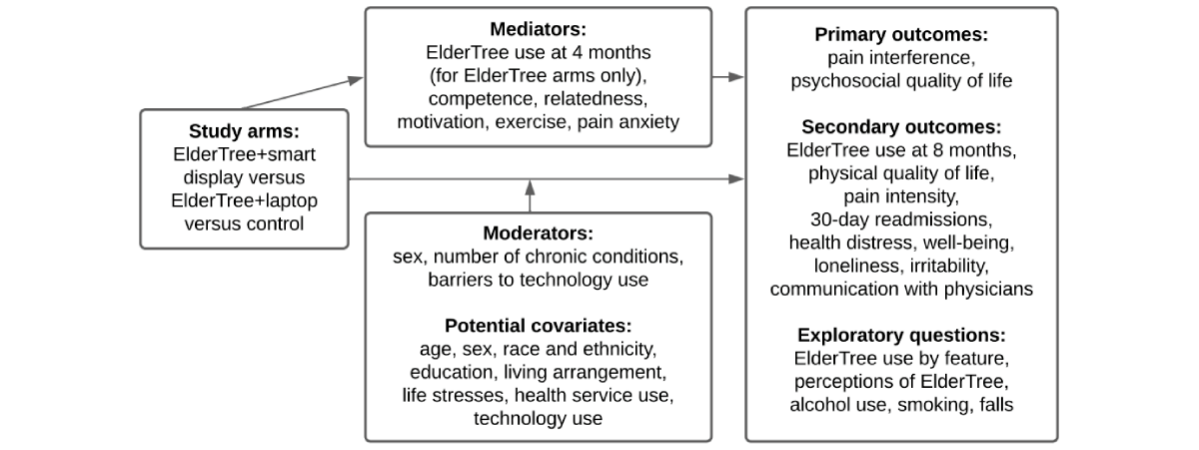
Study logic.

Figure 2 illustrates the study logic. Our primary outcome is the differences between patients in the ET-SD arm, patients in the ET-LT arm, and control patients in pain interference and psychosocial quality of life.

#### Secondary Outcomes

There are several secondary outcomes. First, we will determine whether ET-SD, relative to ET-LT, increases participants’ use of the intervention at 8 months. The original trial of ElderTree found high and sustained engagement relative to reports for other health apps [36]. Nonetheless, many participants did not use it extensively, a problem shared by all digital apps [59-62]. Thus, overcoming barriers to sustained, in-depth use is a critical challenge. This study is designed to investigate whether the voice-controlled interface, relative to the laptop, increases adoption and sustains the use of the intervention, thereby improving its effects on quality of life and health outcomes.

Outcomes also include differences between all 3 groups on measures of physical quality of life, well-being, communication with health care providers, pain intensity and duration, 30-day hospital readmission rates, health distress, loneliness, and irritability. In addition, we will compare ET-LT with the control group, given the unique focus on chronic pain in this study.

#### Exploratory Questions

We will measure the level of use of each ElderTree feature in both platforms and assess use differences between the 2 ElderTree arms as well as participants’ perceptions of the intervention. Differences between the ET-SD, ET-LT, and control groups on alcohol use, smoking, and falls will also be explored.

#### Mediation

For the 2 ElderTree arms, we will investigate whether the effects of study arm on changes from baseline to end point in primary and secondary outcomes are mediated by use of the intervention at the 4-month midpoint. For all study arms, we will also investigate whether feelings of competence, social relatedness, and intrinsic motivation as well as exercise and pain anxiety mediate the effects of the arm on all outcomes.

#### Moderation

We will investigate whether the effects of study arm on changes from baseline to end point in primary and secondary outcomes are moderated. That is, analyses will test whether the benefits of smart displays (vs laptops) vary by participant sex (women will show more benefit than men), number of chronic conditions (patients with more conditions will show more benefit), and number of barriers to using technology (those with fewer barriers will show more benefit).

#### Potential Covariates

We will consider a number of covariates: age, sex, race and ethnicity, education, living arrangement, life stresses, health service use, and technology use. Of this list, those variables that significantly predict our primary outcomes and improve the overall model’s Akaike information criterion will be included in the final analysis.

### Measures

#### Overview

Table 2 lists all planned variables and their measures. Scales have been selected to have good psychometric properties with similar populations. Patient-reported measures, including modifications to validated scales, are described following the table.

**Table 2:** Measures used to evaluate ElderTree on a smart display platform versus ElderTree on a laptop platform versus control.

Variable			Measure		Survey questions, N		Source		Psychometrics
**Primary** **outcomes**									
	Pain interference	PROMIS-29^a^		4		Patient		*α* range ≥.88 [63]	
	Psychosocial quality of life	PROMIS-29		12		Patient		*α* range ≥.90 [64]	
**Secondary outcomes**									
	ElderTree use at 8 months	Number of log-ons		N/A^b^		ElderTree		N/A	
	Physical quality of life	PROMIS-29		12		Patient		*α* range ≥.90 [64]	
	Pain intensity	PROMIS-29		3		Patient		*α* range ≥.90 [64]	
	30-day readmissions	Number of admissions		1		Patient		N/A	
	Health distress	Lorig Health Distress Scale		4		Patient		*α*=.87 [65]	
	Well-being	WHO^c^ Well-Being Index		5		Patient		*α*=.93 [66]	
	Loneliness	NIH^d^ Toolbox		5		Patient		*α*=.93 [67]	
	Irritability	Brief Irritability Test		5		Patient		*α* range ≥.88 [68]	
	Communication with physicians	Lorig Communication with Physicians		3		Patient		*α*=.89 [65]	
**Exploratory questions**									
	ElderTree feature use	Number of page views per feature		N/A		ElderTree		N/A	
	ElderTree perceptions	Created for study; Lund		12		Patient		*α*=.91 [69,70]	
	Alcohol use	Alcohol Use Disorders Identification Test		3		Patient		*α*=.69-.91 [71]	
	Smoking	Number of cigarettes per day		1		Patient		N/A	
	Falls	Number of falls in last 6 months		2		Patient		N/A	
**Mediator candidates**									
	ElderTree use at 4 months	Number of page views		N/A		ElderTree		N/A	
	Competence	Self-Efficacy for Managing Chronic Disease		6		Patient		*α*=.91 [72]	
	Relatedness	McTavish Bonding Scale		5		Patient		*α*=.85 [50]	
	Motivation	Treatment Self-Regulation Questionnaire		4		Patient		*α*=.85 [73]	
	Exercise	Created for study		4		Patient		N/A	
	Pain anxiety	Pain Catastrophizing Scale		13		Patient		*α*=.87 [74]	
**Moderator candidates**									
	Sex	Sex		1		Patient		N/A	
	Chronic conditions	Total number of conditions		16		Patient		N/A	
	Barriers to technology use	Created for study		2		Patient		N/A	
**Potential covariates**									
	Demographics	Age, sex, race and ethnicity, education, and living arrangement		6		Patient		N/A	
	Other	Life stresses, health service use, and technology use		3		Patient		N/A	

^a^PROMIS-29: Patient-Reported Outcomes Measurement Information System–29.

^b^N/A: not applicable.

^c^WHO: World Health Organization.

^d^NIH: National Institutes of Health.

#### Primary and Secondary Outcomes

Pain interference will be assessed using 4 items from the Patient-Reported Outcomes Measurement Information System (PROMIS-29) global health measure (version 2.1) [63,64] asking how much the patients’ pain has interfered with day-to-day activities, work around the home, ability to participate in social activities, and household chores. Psychosocial quality of life will be assessed using 12 PROMIS-29 items asking about anxiety, depression, and the ability to participate in various roles and activities.

ElderTree use at 8 months will be measured by the number of log-ons to the system. Physical quality of life will be assessed using PROMIS-29 items asking patients to rate physical functions such as ability to do chores and use stairs, fatigue, and sleep quality over the last 7 days. Pain intensity will be measured using a PROMIS-29 item on average intensity of pain in the last week and an item from Lorig on intensity of pain at its worst over the last 7 days. An additional item from Lorig will ask about the duration of pain over the last 7 days [65]. Participants will report 30-day hospital readmissions for or as a consequence of the same problem within the last 4 months. Health distress (discouragement, fear, worry, and frustration regarding health and health problems) will be measured using the Lorig Health Distress Scale [75]. Well-being in the last 7 days will be measured using 5 items from the World Health Organization Well-Being Index [66]; loneliness will be measured using 5 items from the National Institutes of Health Toolbox Social Relationship Scale, Loneliness subscale [76]; and irritability in the last 7 days will be measured using the 5-item Brief Irritability Test [68]. Finally, communication with health care providers will be assessed using the Lorig Communication with Physicians measure [75].

#### Exploratory Questions

ElderTree use data, including features used and duration of use, will be collected automatically. At baseline, 4 months, and 8 months, patients will report on alcohol use in the last 4 months with 3 consumption items from the Alcohol Use Disorders Identification Test [71] and on smoking with average number of cigarettes per day. At baseline and 8 months, falls will be assessed with 2 items asking how often the participant has fallen in the last 6 months and how many falls required medical attention. A fall is defined in the survey as “the body going to the ground without being pushed.” Finally, for the purposes of future development, participants in the 2 ElderTree groups and clinicians will be asked about their perceptions of the system.

#### Mediation

ElderTree use at 4 months will be measured as the number of page views per service. Feelings of competence will be assessed using the 6-item Self-Efficacy for Managing Chronic Disease scale, which asks about patients’ confidence in, for example, keeping “fatigue caused by your conditions from interfering with the things you want to do.” The time frame and ranking scale of the questions will be modified to align with our other ranked measures and the timing of the patient surveys [72,77]. Relatedness will be assessed with the 5-item McTavish Bonding Scale, in which patients indicate the frequency of particular types of support such as “someone you can count on to listen to you when you need to talk” [78]. Motivation will be assessed using 2 items from the autonomous subscale and 2 items from the external regulation subscale of the Treatment Self-Regulation Questionnaire (eg, “I tried to manage my health conditions because I feel pressure from others to do so”) [79]. Participants will respond to 4 items asking about the amount of aerobic, stretching and flexibility, strength, and balance exercises performed per week. For pain anxiety, patients will rank 13 items from the Pain Catastrophizing Scale [74].

#### Moderation

The patients will indicate their sex. For barriers to technology use, they will indicate the degree of difficulty they have with computers, tablets, and smart speakers as a result of poor vision, poor hearing, problems vocalizing, lack of knowledge, memory problems, and a custom *other*. The total number of chronic conditions will be determined by the participants responding *yes* or *no* to a list of common conditions [18] during eligibility screening and on the 4- and 8-month surveys. Each time point’s count will be used as the moderator for the effect of study arm at that time point.

#### Potential Covariates

Participants will report their age, sex, race and ethnicity, education level, and living arrangement at baseline. At baseline, 4 months, and 8 months, they will complete checklists of life stresses in the last 4 months, health services used in the last 4 months, and technology use in the last 4 months.

### Data Analysis

Our primary outcome used to power the study is reduction in pain interference. On the basis of PROMIS validation studies with chronic pain samples, a difference of ≥3 points between study arms was considered to indicate a clinically meaningful difference [80]. The PROMIS website also provides the mean and SD of the *T*-score metric (mean 50, SD 10). We powered the analysis to be able to detect a 3-point difference (an effect size of *d*=0.30) between the control and ET-LT groups, and then the same magnitude of effect between the ET-LT and ET-SD groups (an effect size of *d*=0.60 for control vs ET-SD). Across 10,000 linear mixed model simulations, a postattrition N=255, we would have power of >80% to detect the study arm × time interaction. In a previous ElderTree trial, 353 (90.5%) of 390 participants completed the 6-month survey, and 310 (79.5%) of 390 completed the 12-month survey [36]. In the completed surveys, data were missing for approximately 2% of the core items. We expect similar rates in this study, or approximately 87% survey completion at 8 months. Thus, we increased the total sample size after attrition to 291 to increase the likelihood of detecting effects on the outcomes with 2 moderators: sex and number of chronic conditions.

### Data Collection Methods

#### Patient Surveys

The following patient-reported measures will be gathered via participant surveys at baseline, 4 months, and 8 months: psychosocial quality of life, physical quality of life, pain, 30-day hospital readmissions, health distress, well-being, loneliness, irritability, communication with physicians, alcohol use, smoking, falls, barriers to technology use, life stresses, health service use, technology use, competence, relatedness, motivation, exercise, and pain anxiety. At 4 and 8 months, participants will respond to the same checklist of common chronic conditions, including pain, that they answered at eligibility screening. Demographics will be gathered only at baseline. The baseline, 4-month, and 8-month surveys containing all questions posed to participants are provided in Multimedia Appendix 1.

Surveys will be mailed to patients in all groups with a self-addressed stamped envelope and will take 20 to 30 minutes to complete. Patients will be phoned if they do not respond after 2 weeks. Patients can contact the study staff for more details if questions arise. Contact with patients can be requested by the staff if data integrity or compliance issues are detected in the ongoing data review. Survey data will be entered into REDCap. Participants in the 2 ElderTree arms will be paid US $10 to complete each of the 3 surveys (US $30 in total), and those in the control group will be paid US $30 to complete each of the 3 surveys (US $90 in total).

#### ElderTree System Data

Data from the ElderTree weekly survey will be used to assess medication adherence; falls; thinking and memory; mood; healthy meals, snacks, and drinks; physical activity; quality time with others; sleep; pain; and balance. For the ElderTree system use outcomes, keystrokes for ET-LT, voice commands for ET-SD, and time on the system will be collected continuously. Data on ElderTree use in both arms will be collected in time-stamped log files. The primary measure of use will be log-ons per week. Additional use measures will be number of ElderTree features used, pages viewed, messages posted, and weekly surveys completed.

#### Qualitative Interviews

Interviews will be conducted using prepared scripts by people unaffiliated with the study, trained and monitored by one of the co–principal investigators (MLM). Data will be gathered from 32 patients, 16 (50%) in each of the 2 ElderTree arms, balanced by clinic versus community recruitment site and by number of chronic conditions (≤5 vs ≥6). Half of each group will be interviewed at 4 months, and the other half will be interviewed at 8 months. This balances the need for insight into patient experience with the need to avoid confounding interview effects with ElderTree effects. They will be asked about insights critical to the ultimate dissemination of interventions such as ElderTree: barriers to use, technical issues that arose, whether and how ElderTree fit into their day, what could be done to make ElderTree better, reactions to the device, and whether and how ElderTree came up in appointments with their physician.

Interviews are expected to last 30 to 60 minutes and are based on a standard set of questions, although clarification questions may vary. Potential participants will be contacted by ElderTree system messaging or by phone. All interviews will be transcribed for more detailed coding, including quantitative tagging of key concepts.

Clinicians will encounter ElderTree only through the clinician report. We will record and analyze clinicians’ comments and questions during meetings where we introduce the study and the clinician report to the clinics, and we will ask about anticipated barriers and benefits. We will document the protocols that clinics use to receive and disseminate the report to clinicians. We will interview 5 clinicians at the end of data collection about their experiences with and perceptions of the report. Interviews are expected to last 10 to 20 minutes and are based on a standard set of questions, although clarification questions may vary.

### Retention

We will promote retention by providing ready access to support for patients’ use of the technologies and by actively following up with patients to encourage them to return surveys. If a survey is not returned within 2 weeks, a research team member will call to check that the survey was received and encourage the patient to complete and return it in the addressed stamped envelope. The date and time of the phone call will be recorded in REDCap along with whether the researcher talked to the participant directly or left a message and any information gathered during the phone conversation. If we cannot reach the participant, another copy of the survey will be sent with a personal note asking them to complete it or call us on our toll-free number if they have questions or are no longer interested. In our study of older adults with MCCs, for which we have recently completed data collection, survey response rates were 94.7% at 6 months, 93.6% at 12 months, and 92.3% at 18 months [37].

### Data Management

To mitigate the risk of breaches of patient confidentiality, all participants are assigned a unique code number. All contact information and survey data are housed electronically in REDCap. Survey data are double-entered by 2 different individuals to ensure accuracy. Paper-based files are stored in a locked room in locked file cabinets and can be accessed only by authorized personnel. The database administrator provides access to study data at appropriate levels for various members of the research team. Members of the research team are able to view deidentified individual and clinic-level aggregations of the variables.

### Statistical Methods

#### Predictor Assumptions

Successful randomization of participants will be tested based on sex, recruitment site (UW Health, Access Centers, and the community), and number of chronic conditions. If randomization fails for any of these variables, it will be added as a covariate in subsequent analyses. We will test to see if the nesting structure of recruitment site significantly influences our findings. If data cannot be pooled across sites, site will be addressed either by multilevel modeling or by treating recruitment site as a moderator, depending on the analyses being run.

#### Outcome Assumptions

Normality, linearity, and homoscedasticity and homogeneity of variance for the outcome data will be assessed using descriptive statistics and graphical representations. Data transformation, linear mixed models, or nonparametric tests will be used to deal with assumption failures of the outcome data.

#### Missing Data

In previous work with older adults using ElderTree, we kept the missing data on core interview items to approximately 2%; we expect similar rates in this study. In primary care, data are not likely to be missing at random; that is, the probability that data are missing relates to what the data would have been had the data been observed. We will conduct a sensitivity analysis on missing data using logistic regression to examine whether dropout at follow-up is associated with observed or assigned factors, covariates, or outcomes at baseline [81]. If missing data affect power or are significantly not missing at random, linear mixed models or multiple imputation will be used [82]. If patients drop out, we will not use any of their data beyond their withdrawal date. Data before the withdrawal date will be included in the intention-to-treat analysis.

#### Effectiveness of Control Versus ET-LT Versus ET-SD

Linear mixed-effects models, which account for dependence among successive observations for the same participant and can address incomplete data, will be used to examine the effects of study arm (ET-SD vs ET-LT vs control, a between-participant factor) on our outcomes over time. We will conduct specific treatment×time contrasts both between and within groups to test time-based effects. For binary, count, and other nonnormal data, generalized mixed-effects models will be used.

#### Mediation and Moderation Effects

Structural equation modeling will explore the effects of mediation on the relationship between study arms and our 2 primary outcomes. We anticipate that the impact of study arm on pain interference and psychological quality of life will be mediated by our theoretical constructs of competence, relatedness, and motivation; exercise; and pain anxiety at 4 months. Structural equation models involving ElderTree use will be run separately only for the ET-SD and ET-LT arms. We anticipate that sex, number of chronic conditions, and barriers to technology use will moderate the indirect paths of the mediation models.

#### Impact of Interim Analyses on Type 1 Error for Whole-Sample Analysis

Findings that are significant in the interim but not the final analyses will be treated as nonsignificant. The final analysis will examine all data over time. For analyses that address the same hypothesis, a Holm–Bonferroni correction will be applied.

#### Qualitative Analysis

For data sets of patient and physician interviews, a coding scheme of key themes will be constructed based on the research questions of perceived benefits and barriers to use and examination of the data. Each scheme will be pilot-tested, and then 2 trained coders will code an overlapping subsample of 20% of content. Once reliability is established with a minimum Krippendorff *α* of .80 per category, the coders will work independently to code the rest of the material.

### Ethics Approval

This study protocol received ethical approval from the University of Wisconsin Health Sciences and Minimal Risk Research Institutional Review Board (reference 2020-0868). All amendments to the protocol have been submitted to the institutional review board and approved. This study complies with the Declaration of Helsinki and its later amendments.

## Results

Recruitment began in August 2021 and will run through April 2023. The intervention period will end in December 2023. As of March 21, 2022, a total of 109 participants have been recruited. The findings will be disseminated via peer-reviewed publications.

## Discussion

### Study Overview

To our knowledge, this is the first study to examine with a large sample and a long time frame whether a smart device can, in reality, perform as well as or better than a laptop in implementing a health intervention. To assess this, we will examine group differences in pain and quality of life measures among patients in high need of resources to help manage their chronic conditions. A 2021 systematic review of research on the use of “voice-based conversational agents for the prevention and management of chronic conditions” concluded that the field is “in its infancy” [83].

### Comparison With Prior Work

Our center is well-positioned to pursue this question as we have an evidence-based laptop system available for adaptation and comparison. The results of our first trial of ElderTree indicated that older patients coping with multiple comorbidities were most likely to benefit from such an intervention [36], and our subsequent study is testing that hypothesis [37]. The study described in this protocol, targeting similar patients, is designed to test and compare how delivery platform affects pain and psychosocial variables that have shown improvement with the laptop version [36]. Comparing platforms with this highly challenged population may highlight more starkly the relative advantages and pitfalls of voice-controlled platforms for eHealth delivery.

### Future Directions

Applications for voice-controlled devices are in the early stages of development. Some known limitations, such as problems with dictation and word recognition, are certain to improve. Others, including the lack of ability to review longer lists of results or read larger amounts of text, may be endemic. Still other limitations, as yet unknown and coming to light as developers stumble upon them, may or may not be resolved. Whether the advantages of the technology outweigh the disadvantages and how its unique capabilities may be optimized, if at all, are empirical questions to be explored in this and future studies.

A specific question is whether smart devices for eHealth may be more effective depending on the health context. For example, this protocol describes an intervention targeting chronic pain. We are also in the system development stage of a trial of smart devices funded by the National Heart, Lung and Blood Institute in which the primary outcome is functional health, a variable that includes multiple physical and psychoemotional factors.

### Conclusions

The goal of ElderTree, regardless of delivery platform, is to improve patient self-management and provide convenient, ongoing support as a means to improve health and quality of life. At the same time, and as a result, interventions such as ElderTree may help relieve some of the burden on the primary health care system. As older patients with MCCs are such a large and growing cohort, the implications for access and cost as well as patient well-being are significant. Making the best use of current and developing technologies is a critical part of this effort.

## Appendix

Multimedia Appendix 1 Baseline, 4-month, and 8-month patient surveys.

Multimedia Appendix 2 Peer review from NCI-J - Healthcare Information Technology Research [HITR] Study Section, Agency for Healthcare Research and Quality (Agency for Healthcare Research and Quality, USA).

## Abbreviations

CHESS: Comprehensive Health Enhancement Support System
CONSORT: Consolidated Standards of Reporting Trials
ET-LT: ElderTree on a laptop platform
ET-SD: ElderTree on a smart display platform
MCCs: multiple chronic conditions
PROMIS-29: Patient-Reported Outcomes Measurement Information System–29
REDCap: Research Electronic Data Capture
UW Health: University of Wisconsin–Madison Department of Family Medicine and General Internal Medicine system
