# Supplementary material for: Using Smart Displays to Implement an eHealth System for Older Adults With Multiple Chronic Conditions: Protocol for a Randomized Controlled Trial
Source: JMIR Res Protoc. 2022 May 5;11(5):e37522. doi: 10.2196/37522 (PMC9121223; doi:10.2196/37522)
Supplement: Multimedia Appendix 1 [file resprot_v11i5e37522_app1.pdf]

Recruitment ID#: _____________

Study ID#: _____________

Today's Date: ______________

ElderTree Smart System Baseline Survey

If you have any questions please contact:

[study coordinator]
[email]
University of Wisconsin – Madison
Center for Health Enhancement Systems Studies
1513 University Avenue
Madison, WI 53706

[tech support and coordinator phones]

Thank you for taking part in this study.

Your answers on this survey are important to us.

Any information you share is confidential.

If you feel uncomfortable with any question, you can skip it.

You are free to stop filling out the survey at any time.

When you are finished with the survey,
please return in the stamped address envelope.

INSTRUCTIONS:

Please check one box per question or row unless otherwise indicated.
Additional instructions are included in brackets [ ].

1. **In the <u>past 4 months</u>, how much (if at all) did any of these issues make it hard for you to use a *<u>computer or iPad</u>*? [If you never use a computer or iPad, check "not applicable."]**

	**Not at all**	**Slightly**	**Moderately**	**Very**	**Extremely**	**Not applicable**
Vision (even with glasses)	❑	❑	❑	❑	❑	❑
Hearing (even with hearing aids)	❑	❑	❑	❑	❑	❑
Voice Issues (hard to speak clearly)	❑	❑	❑	❑	❑	❑
Lack of knowledge of how to use it	❑	❑	❑	❑	❑	❑
Memory	❑	❑	❑	❑	❑	❑
Other (please specify): ____________________	❑	❑	❑	❑	❑	❑

2. **In the <u>past 4 months</u>, how much (if at all) did any of these issues make it hard for you to use a *<u>smart speaker like Alexa, Google Home, or Amazon Echo</u>*? If you never use a smart speaker, check "not applicable."**

	**Not at all**	**Slightly**	**Moderately**	**Very**	**Extremely**	**Not applicable**
Vision (even with glasses)	❑	❑	❑	❑	❑	❑
Hearing (even with hearing aids)	❑	❑	❑	❑	❑	❑
Voice Issues (hard to speak clearly)	❑	❑	❑	❑	❑	❑
Lack of knowledge of how to use it	❑	❑	❑	❑	❑	❑
Memory	❑	❑	❑	❑	❑	❑
Other (please specify): ____________________	❑	❑	❑	❑	❑	❑

3. In the past 4 months, how often did you use each the following?

	I don't know what this is/never use	Rarely	Sometimes	Often	Very often
Zoom, Skype, or other video chat	❑	❑	❑	❑	❑
Email	❑	❑	❑	❑	❑
Facebook	❑	❑	❑	❑	❑
Texting	❑	❑	❑	❑	❑
Alexa or similar device	❑	❑	❑	❑	❑

4. In the past 4 months, have you used a wearable device such as a FitBit or Apple Watch capable of tracking your physical activity?

❑ Yes

❑ No (If No, skip to Question #5)

If Yes, which device(s) have you used?

____

	Not helpful					Very helpful
**How helpful did you find the wearable device(s)?**	❑0	❑1	❑2	❑3	❑4	❑5

	Not easy					Very easy
**How easy was it to use the wearable device(s)?**	❑0	❑1	❑2	❑3	❑4	❑5

If you have stopped using the device(s), why?

____

____

5. In the past 4 months, how many visits have you made to each of the following (Include visits in person, by phone, or video)? [If none, write 0.]

*We know it's hard to remember these details for the past 4 months - do the best you can. We really appreciate it.

______ Primary care (e.g., primary care doctor, physician assistant, nurse practitioner)

______ Specialist doctors (e.g., heart, lung, diabetes, cancer, ear/nose/throat, sleep, arthritis, bladder/kidney, women's issues)

______ Surgeon

______ Physical therapist, occupational therapist, chiropractor

______ Counselor, psychologist, psychiatrist, addiction treatment specialist

______ Ophthalmologist or eye doctor

______ Dentist

______ Lab visits (e.g., blood draws, mammogram screening, X-ray, radiation)

______ Urgent care clinic

______ Emergency room

6. A key issue for hospitals is if patients are readmitted within 30 days for the same health problem or complications of that problem. Did that happen to you in the past four months?

- ❑ Yes
- ❑ No

If Yes, how many times? ________________

7. We would like to know how confident you are in doing certain activities, given your chronic health conditions. Please check the box that corresponds to your confidence that you have been able to do the following tasks regularly in the past 4 months.

**In the past 4 months, how confident have you felt that you could…**	**Not at all confident**	**A little confident**	**Somewhat confident**	**Mostly confident**	**Totally confident**
Keep the fatigue caused by your conditions from interfering with the things you want to do?	❑	❑	❑	❑	❑
Keep the physical discomfort or pain of your conditions from interfering with the things you want to do?	❑	❑	❑	❑	❑
Keep the emotional distress caused by your conditions from interfering with the things you want to do?	❑	❑	❑	❑	❑
Keep any other symptoms or health problems you have from interfering with the things you want to do?	❑	❑	❑	❑	❑
Do the different tasks and activities needed to manage your health conditions so as to reduce your need to see a doctor?	❑	❑	❑	❑	❑
Do things other than just taking medication to reduce how much your conditions affect your everyday life?	❑	❑	❑	❑	❑

**8. In the <u>past 4 months</u>, I tried to manage my health conditions because…**	**Strongly Disagree**	**Disagree**	**Neither agree or disagree**	**Agree**	**Strongly Agree**
Others would be upset with me if I did not	❑	❑	❑	❑	❑
I want to take responsibility for my own health	❑	❑	❑	❑	❑
It is an important choice I really want to make	❑	❑	❑	❑	❑
I feel pressure from others to do so	❑	❑	❑	❑	❑

**9. In the <u>past 4 months</u>, when I was in pain…**	**Not at all**	**A little bit**	**Somewhat**	**Quite a bit**	**Very much**
I worried all the time about whether the pain will end.	❑	❑	❑	❑	❑
I felt I couldn't go on.	❑	❑	❑	❑	❑
It was terrible and I thought it was never going to get any better.	❑	❑	❑	❑	❑
It was awful and I felt that it overwhelmed me.	❑	❑	❑	❑	❑
I felt I couldn't stand it anymore.	❑	❑	❑	❑	❑
I became afraid that the pain would get worse.	❑	❑	❑	❑	❑
I kept thinking of other painful events.	❑	❑	❑	❑	❑
I anxiously wanted the pain to go away.	❑	❑	❑	❑	❑
I couldn't seem to keep it out of my mind.	❑	❑	❑	❑	❑
I kept thinking about how much it hurt.	❑	❑	❑	❑	❑
There was nothing I could do to reduce the intensity of the pain.	❑	❑	❑	❑	❑
I kept thinking about how badly I wanted the pain to stop.	❑	❑	❑	❑	❑
I wondered whether something serious might happen.	❑	❑	❑	❑	❑

**10. In the <u>past 4 months</u>, how often has there been someone…**	**Never**	**Rarely**	**Sometimes**	**Often**	**Always**
To give you information if you need it	❑	❑	❑	❑	❑
To give you helpful advice when dealing with a problem	❑	❑	❑	❑	❑
Who makes you feel appreciated	❑	❑	❑	❑	❑
You can count on to listen to you when you need to talk	❑	❑	❑	❑	❑
With whom to share your most private worries and fears	❑	❑	❑	❑	❑

11. Have any of these events caused you to feel significant stress in the <u>past 4 months</u>? [CHECK ALL THAT APPLY]

- ❑ Change in marital or partner status (death, divorce, separation, marriage)
- ❑ Death of a very close friend or family member
- ❑ Change of work status (business failure, layoff, retirement, return to work)
- ❑ Personal injury or illness
- ❑ Change in health of a very close friend or family member
- ❑ Sexual difficulties
- ❑ Change in financial status
- ❑ Loss or change of caregiver
- ❑ Change in living arrangement
- ❑ Change in ability to engage in physical activities
- ❑ Change in religious and/or social activities
- ❑ Change in sleeping habits
- ❑ Legal problems
- ❑ Difficulty with drugs or alcohol
- ❑ None of the above

12. In the past 4 months, when you visited your doctor, how often did you do the following?
[If you did not visit your doctor in the past 4 months check "Not applicable"]

	Never	Almost never	Sometimes	Fairly often	Very often	Always	Not applicable
Prepare a list of questions for your doctor.	❑	❑	❑	❑	❑	❑	❑
Ask questions about the things you want to know and things you don't understand about your treatment.	❑	❑	❑	❑	❑	❑	❑
Discuss any personal problems that may be related to your illness.	❑	❑	❑	❑	❑	❑	❑

13. Please answer the following questions about your alcohol use in the past 4 months.

**In the past 4 months…**	**Never**	**Monthly or less**	**Two to four times a month**	**Two to three times per week**	**Four or more times a week**
**How often did you have a drink containing alcohol?**	❑	❑	❑	❑	❑

[If "Never" skip to #14]

**In the past 4 months…**	**1 or 2 drinks**	**3 or 4**	**5 or 6**	**7 to 9**	**10 or more**
**How may drinks containing alcohol did you have on a typical day when you were drinking?**	❑	❑	❑	❑	❑

In the <u>past 4 months</u>…	Never	Less than monthly	Monthly	Weekly	Daily or almost daily
**How often did you have six or more drinks on one occasion?**	❑	❑	❑	❑	❑

14. On average, <u>in the past 4 months</u>, how many cigarettes did you smoke a day?

____

**15. In the <u>past 4 months</u>, how many days of the week did you do exercises…**	**0 days**	**1 day**	**2 days**	**3 days**	**4 days**	**5 days**	**6 days**	**7 days**
To get your heart rate up or get aerobic exercise (e.g. brisk walk)	❑	❑	❑	❑	❑	❑	❑	❑
For stretching and flexibility	❑	❑	❑	❑	❑	❑	❑	❑
For strength (e.g. weights, therabands, other strength exercises)	❑	❑	❑	❑	❑	❑	❑	❑
For balance (exercises to improve your balance)	❑	❑	❑	❑	❑	❑	❑	❑

For the next question we are asking about falls over the <u>past 6 months</u> (not 4 months).

16. Please answer the follow questions about falls over the <u>past 6 months</u>.

About how many times have you fallen (fall is when your body goes to the ground without being pushed)?	
How many of these falls require medical attention?	

The next two sets of questions ask about the past month.

**17. How much time in the past month…**	**None of the time**	**A little of the time**	**Some of the time**	**A good bit of the time**	**Most of the time**	**All of the time**
Were you discouraged by your health problems?	❑	❑	❑	❑	❑	❑
Were you fearful about your future health?	❑	❑	❑	❑	❑	❑
Was your health a worry in your life?	❑	❑	❑	❑	❑	❑
Were you frustrated by your health problems?	❑	❑	❑	❑	❑	❑

**18. In the past month…**	**Never**	**Rarely**	**Sometimes**	**Usually**	**Always**
I feel alone and apart from others.	❑	❑	❑	❑	❑
I feel left out.	❑	❑	❑	❑	❑
I feel that I am no longer close to anyone.	❑	❑	❑	❑	❑
I feel alone.	❑	❑	❑	❑	❑
I feel lonely.	❑	❑	❑	❑	❑

Thanks so much for answering all of those questions!

The next set of questions ask about your experiences over the past 7 days, so we know what's been going on recently.

**19. During the past 7 days…**	**Without any difficulty**	**With a little difficulty**	**With some difficulty**	**With much difficulty**	**Unable to do**
Are you able to do chores such as vacuuming or yard work?	❑	❑	❑	❑	❑
Are you able to go up and down stairs at a normal pace?	❑	❑	❑	❑	❑
Are you able to go for a walk of at least 15 minutes?	❑	❑	❑	❑	❑
Are you able to run errands and shop?	❑	❑	❑	❑	❑

**20. During the past 7 days…**	**Not at all**	**A little bit**	**Somewhat**	**Quite a bit**	**Very much**
I feel fatigued.	❑	❑	❑	❑	❑
I have trouble starting things because I am tired.	❑	❑	❑	❑	❑
How run-down did you feel on average?	❑	❑	❑	❑	❑
How fatigued were you on average?	❑	❑	❑	❑	❑

**21. In the past 7 days…**	**Very poor**	**Poor**	**Fair**	**Good**	**Very good**
My sleep quality was…	❑	❑	❑	❑	❑

**In the past 7 days…**	**Not at all**	**A little bit**	**Somewhat**	**Quite a bit**	**Very much**
My sleep was refreshing.	❑	❑	❑	❑	❑
I had a problem with my sleep.	❑	❑	❑	❑	❑
I had difficulty falling asleep.	❑	❑	❑	❑	❑

**22. In the past 7 days…**	**Not at all**	**A little bit**	**Somewhat**	**Quite a bit**	**Very much**
How much did pain interfere with your day to day activities?	❑	❑	❑	❑	❑
How much did pain interfere with work around the home?	❑	❑	❑	❑	❑
How much did pain interfere with your ability to participate in social activities?	❑	❑	❑	❑	❑
How much did pain interfere with your household chores?	❑	❑	❑	❑	❑

**23. In the past 7 days…**	**No pain**										**Worst imaginable pain**
How would you rate your pain on **average?**	❑0	❑1	❑2	❑3	❑4	❑5	❑6	❑7	❑8	❑9	❑10
How would you rate your pain **at its WORST?**	❑0	❑1	❑2	❑3	❑4	❑5	❑6	❑7	❑8	❑9	❑10

**24. In the past 7 days…**	**Didn't have any**	**A few minutes**	**Several minutes to an hour**	**Several hours**	**A day or two**	**More than 2 days**
When you had pain, how long did it usually last? (If you have several pains, answer in general.)	❑	❑	❑	❑	❑	❑

25. In the **past 7 days**…	Never	Rarely	Sometimes	Usually	Always
I have trouble doing all of my regular leisure activities with others.	❑	❑	❑	❑	❑
I have trouble doing all of the family activities that I want to do.	❑	❑	❑	❑	❑
I have trouble doing all of my usual work (include work at home).	❑	❑	❑	❑	❑
I have trouble doing all of the activities with friends that I want to do.	❑	❑	❑	❑	❑

26. In the **past 7 days**…	Never	Rarely	Sometimes	Often	Always
I felt fearful.	❑	❑	❑	❑	❑
I found it hard to focus on anything other than my anxiety.	❑	❑	❑	❑	❑
My worries overwhelmed me.	❑	❑	❑	❑	❑
I felt uneasy.	❑	❑	❑	❑	❑

27. In the **past 7 days**…	Never	Rarely	Sometimes	Often	Always
I felt worthless.	❑	❑	❑	❑	❑
I felt helpless.	❑	❑	❑	❑	❑
I felt depressed.	❑	❑	❑	❑	❑
I felt hopeless.	❑	❑	❑	❑	❑

28. In the **past 7 days**…	Never	Rarely	Sometimes	Often	Always
I have been grumpy	❑	❑	❑	❑	❑
I have been feeling like I might snap	❑	❑	❑	❑	❑
Other people have been getting on my nerves	❑	❑	❑	❑	❑
Things have been bothering me more than they normally do	❑	❑	❑	❑	❑
I have been feeling irritable	❑	❑	❑	❑	❑

**29. In the past 7 days…**	**All of the time**	**Most of the time**	**More than half of the time**	**Less than half of the time**	**Some of the time**	**At no time**
I have felt cheerful and in good spirits.	❑	❑	❑	❑	❑	❑
I have felt calm and relaxed.	❑	❑	❑	❑	❑	❑
I have felt active and vigorous.	❑	❑	❑	❑	❑	❑
I woke up feeling fresh and rested.	❑	❑	❑	❑	❑	❑
My daily life has been filled with things that interest me.	❑	❑	❑	❑	❑	❑

This next set of questions will ask you about everyday things you do in your life, and to what extent you feel limited in doing these activities. Please select the one answer that comes closest to the way you have been feeling.

**30. To what extent do you feel limited in…?**	**Not at all**	**A little**	**Somewhat**	**A lot**	**Completely**
Keeping in touch with others	❑	❑	❑	❑	❑
Visiting friends and family in their homes	❑	❑	❑	❑	❑
Providing care or assistance to others	❑	❑	❑	❑	❑
Working at a volunteer job	❑	❑	❑	❑	❑
Taking part in active recreation	❑	❑	❑	❑	❑
Traveling out of town	❑	❑	❑	❑	❑
Inviting people into your home	❑	❑	❑	❑	❑
Going out with others to public places	❑	❑	❑	❑	❑
Taking part in organized social activities	❑	❑	❑	❑	❑

This next set of questions will ask you about your ability to do specific activities as part of your daily routines. We are interested in your *sense of your ability* to do it on a typical day <u>without</u> the help of someone else, and <u>without</u> the use of a cane, walker, or any other assistive walking device (or a wheelchair or scooter).

It is not important that you actually do the activity on a daily basis. In fact, there may be some activities that you don't do at all. You can still answer these questions by assessing how difficult you <u>think</u> they would be for you to do on an average day.

**31. How much difficulty do you have…** (Remember this is without the help of someone else and without the use of any assistive walking device.)	**None**	**A little**	**Some**	**Quite a lot**	**Cannot do**
Unscrewing the lid off a previously unopened jar without using any devices	❑	❑	❑	❑	❑
Going up & down a flight of stairs inside, using a handrail	❑	❑	❑	❑	❑
Putting on and taking off long pants (including managing fasteners)	❑	❑	❑	❑	❑
Running ½ mile or more	❑	❑	❑	❑	❑
Using common utensils for preparing meals (e.g., can opener, potato peeler, or sharp knife)	❑	❑	❑	❑	❑
Holding a full glass of water in one hand	❑	❑	❑	❑	❑
Running a short distance, such as to catch a bus	❑	❑	❑	❑	❑
Reaching overhead while standing, as if to pull a light cord	❑	❑	❑	❑	❑
Sitting down in and standing up from a low, soft couch	❑	❑	❑	❑	❑
Putting on and taking off a coat or jacket	❑	❑	❑	❑	❑
Reaching behind your back as if to put a belt through a belt loop	❑	❑	❑	❑	❑
Rip open a package of snack food (e.g. cellophane wrapping on crackers) using only your hands	❑	❑	❑	❑	❑
Pouring from a large pitcher	❑	❑	❑	❑	❑
Getting into and out of a car/taxi (sedan)	❑	❑	❑	❑	❑
Hiking a couple of miles on uneven surfaces, including hills	❑	❑	❑	❑	❑

	None	A little	Some	Quite a lot	Cannot do
Going up and down 3 flights of stairs inside, using a handrail	❑	❑	❑	❑	❑
Picking up a kitchen chair and moving it, in order to clean	❑	❑	❑	❑	❑
Using a step stool to reach into a high cabinet	❑	❑	❑	❑	❑
Making a bed, including spreading and tucking in bed sheets	❑	❑	❑	❑	❑
Carrying something in both arms while climbing a flight of stairs (e.g. laundry basket)	❑	❑	❑	❑	❑
Bending over from a standing position to pick up a piece of clothing from the floor	❑	❑	❑	❑	❑
Getting up from the floor (as if you were laying on the ground)	❑	❑	❑	❑	❑
Washing dishes, pots, and utensils by hand while standing at sink	❑	❑	❑	❑	❑
Stepping on and off a bus	❑	❑	❑	❑	❑

Please answer the following questions <u>if you do NOT use a cane, walker, or other walking device</u>.

[If you use a walking device, skip to #33]

**32. How much difficulty do you have…** (Remember this is without the help of someone else and without the use of any assistive walking device.)	None	A little	Some	Quite a lot	Cannot do
Walking a mile, taking rests as necessary	❑	❑	❑	❑	❑
Going up & down a flight of stairs outside, without using a handrail	❑	❑	❑	❑	❑
Stepping up and down from a curb	❑	❑	❑	❑	❑
Opening a heavy, outside door	❑	❑	❑	❑	❑
Walking around on floor of your home, taking into consideration thresholds, doors, furniture, and a variety of floor coverings	❑	❑	❑	❑	❑
Walking several blocks	❑	❑	❑	❑	❑
Taking a 1 mile, brisk walk without stopping to rest	❑	❑	❑	❑	❑
Walking on a slippery surface, outdoors	❑	❑	❑	❑	❑

Please answer the following questions <u>if you DO use a cane, walker, or other walking device</u>.

[If you do not use a walking device, skip to #34]

**33. When you use your cane, walker, or other walking device, how much difficulty do you have…?**	**None**	**A little**	**Some**	**Quite a lot**	**Cannot do**
Walking a mile, taking rests as necessary	❑	❑	❑	❑	❑
Going up & down a flight of stairs outside, without using a handrail	❑	❑	❑	❑	❑
Stepping up and down from a curb	❑	❑	❑	❑	❑
Opening a heavy, outside door	❑	❑	❑	❑	❑
Walking around on floor of your home, taking into consideration thresholds, doors, furniture, and a variety of floor coverings	❑	❑	❑	❑	❑
Walking several blocks	❑	❑	❑	❑	❑
Taking a 1 mile, brisk walk without stopping to rest	❑	❑	❑	❑	❑
Walking on a slippery surface, outdoors	❑	❑	❑	❑	❑

<u>DEMOGRAPHICS</u>

34. Check what applies to you:

❑ Female
❑ Male
❑ Self-describe _____________________________

35. What is your age? _________

36. Are you of Hispanic or Latino origin or descent?

❑ Yes, Hispanic or Latino
❑ No

37. What is your race? [Check all that apply]

- ❑ White
- ❑ Black or African American
- ❑ Asian
- ❑ Native Hawaiian or other Pacific Islander
- ❑ American Indian or Alaskan Native
- ❑ Other, please specify ____

38. What is the highest grade or level of education you have completed?

- ❑ Elementary school
- ❑ Middle school
- ❑ High school
- ❑ Vocational or technical school
- ❑ Some college
- ❑ College graduate
- ❑ Post graduate or professional
- ❑ Other _______________

39. What is your household income?

- ❑ Less than $12,000 a year
- ❑ $12,000-$24,999 a year
- ❑ $25,000-$49,999 a year
- ❑ $50,000-$74,999 a year
- ❑ $75,000 or above
- ❑ Other _______________

40. What type of health insurance do you have? [CHECK ALL THAT APPLY]

- ❑ Medicare
- ❑ Medicare supplemental insurance (e.g., Medicare Advantage Plan, Part B, Part D)
- ❑ Medicaid
- ❑ ObamaCare (Affordable Care Act)
- ❑ Military
- ❑ Private insurance
- ❑ Covered under someone else's policy
- ❑ No insurance
- ❑ Other _______________

Please continue to last page

41. Do you have a significant other? (spouse, partner)

- ❑ Yes
- ❑ No

42. Which of the following best describes your living arrangement?

- ❑ Live in a place that I own
- ❑ Live in a place that I rent
- ❑ Live in an assisted living facility
- ❑ Live in the home or apartment of my son, daughter, other family, or friends
- ❑ Live in a residential care facility where I have my own stove
- ❑ Live in a nursing home
- ❑ Other ____

43. Does anyone else live with you?

- ❑ No
- ❑ Yes → If so, who lives with you? [CHECK ALL THAT APPLY]
    - ❑ Spouse/partner
    - ❑ Son or daughter
    - ❑ Other family members or friends
    - ❑ Paid caregiver
    - ❑ Other ____

Thank you for completing this survey.

Recruitment ID#: _____________

Study ID#: _____________

Today's Date: _______________

ElderTree Smart System

4 Month Survey

If you have any questions please contact:

[study coordinator]
[email]
University of Wisconsin – Madison
Center for Health Enhancement Systems Studies
1513 University Avenue
Madison, WI 53706

[tech support and coordinator phones]

Thank you for taking part in this study.

Your answers on this survey are important to us.

Any information you share is confidential.

If you feel uncomfortable with any question, you can skip it.

You are free to stop filling out the survey at any time.

When you are finished with the survey,
please return in the stamped address envelope.

INSTRUCTIONS:

Please check one box per question or row unless otherwise indicated.
Additional instructions are included in brackets [ ].

1. In the <u>past 4 months</u>, how much (if at all) did any of these issues make it hard for you to use a *<u>computer or iPad</u>*? [If you never use a computer or iPad, check "not applicable."]

	Not at all	Slightly	Moderately	Very	Extremely	Not applicable
Vision (even with glasses)	❑	❑	❑	❑	❑	❑
Hearing (even with hearing aids)	❑	❑	❑	❑	❑	❑
Voice Issues (hard to speak clearly)	❑	❑	❑	❑	❑	❑
Lack of knowledge of how to use it	❑	❑	❑	❑	❑	❑
Memory	❑	❑	❑	❑	❑	❑
Other (please specify): ____________________	❑	❑	❑	❑	❑	❑

2. In the <u>past 4 months</u>, how much (if at all) did any of these issues make it hard for you to use a *<u>smart speaker like Alexa, Google Home, or Amazon Echo</u>*? If you never use a smart speaker, check "not applicable."

	Not at all	Slightly	Moderately	Very	Extremely	Not applicable
Vision (even with glasses)	❑	❑	❑	❑	❑	❑
Hearing (even with hearing aids)	❑	❑	❑	❑	❑	❑
Voice Issues (hard to speak clearly)	❑	❑	❑	❑	❑	❑
Lack of knowledge of how to use it	❑	❑	❑	❑	❑	❑
Memory	❑	❑	❑	❑	❑	❑
Other (please specify): ____________________	❑	❑	❑	❑	❑	❑

3. In the past 4 months, how often did you use each the following?

	I don't know what this is/never use	Rarely	Sometimes	Often	Very often
Zoom, Skype, or other video chat	❑	❑	❑	❑	❑
Email	❑	❑	❑	❑	❑
Facebook	❑	❑	❑	❑	❑
Texting	❑	❑	❑	❑	❑
Alexa or similar device	❑	❑	❑	❑	❑

4. In the past 4 months, how many visits have you made to each of the following (Include visits in person, by phone, or video)? [If none, write 0.]

*We know it's hard to remember these details for the past 4 months - do the best you can. We really appreciate it.

______ Primary care (e.g., primary care doctor, physician assistant, nurse practitioner)

______ Specialist doctors (e.g., heart, lung, diabetes, cancer, ear/nose/throat, sleep, arthritis, bladder/kidney, women's issues)

______ Surgeon

______ Physical therapist, occupational therapist, chiropractor

______ Counselor, psychologist, psychiatrist, addiction treatment specialist

______ Ophthalmologist or eye doctor

______ Dentist

______ Lab visits (e.g., blood draws, mammogram screening, X-ray, radiation)

______ Urgent care clinic

______ Emergency room

5. **A key issue for hospitals is if patients are <u>readmitted within 30 days</u> for the same health problem or complications of that problem. Did that happen to you in the <u>past four months</u>?**
   - ❑ Yes
   - ❑ No

If Yes, how many times? _______________

6. We would like to know how confident you are in doing certain activities, given your chronic health conditions. Please check the box that corresponds to your confidence that you have been able to do the following tasks regularly in the <u>past 4 months</u>.

**In the <u>past 4 months</u>, how confident have you felt that you could…**	**Not at all confident**	**A little confident**	**Somewhat confident**	**Mostly confident**	**Totally confident**
Keep the fatigue caused by your conditions from interfering with the things you want to do?	❑	❑	❑	❑	❑
Keep the physical discomfort or pain of your conditions from interfering with the things you want to do?	❑	❑	❑	❑	❑
Keep the emotional distress caused by your conditions from interfering with the things you want to do?	❑	❑	❑	❑	❑
Keep any other symptoms or health problems you have from interfering with the things you want to do?	❑	❑	❑	❑	❑
Do the different tasks and activities needed to manage your health conditions so as to reduce your need to see a doctor?	❑	❑	❑	❑	❑
Do things other than just taking medication to reduce how much your conditions affect your everyday life?	❑	❑	❑	❑	❑

**7. In the <u>past 4 months</u>, I tried to manage my health conditions because…**	**Strongly Disagree**	**Disagree**	**Neither agree or disagree**	**Agree**	**Strongly Agree**
Others would be upset with me if I did not	❑	❑	❑	❑	❑
I want to take responsibility for my own health	❑	❑	❑	❑	❑
It is an important choice I really want to make	❑	❑	❑	❑	❑
I feel pressure from others to do so	❑	❑	❑	❑	❑

**8. In the <u>past 4 months</u>, when I was in pain…**	**Not at all**	**A little bit**	**Somewhat**	**Quite a bit**	**Very much**
I worried all the time about whether the pain will end.	❑	❑	❑	❑	❑
I felt I couldn't go on.	❑	❑	❑	❑	❑
It was terrible and I thought it was never going to get any better.	❑	❑	❑	❑	❑
It was awful and I felt that it overwhelmed me.	❑	❑	❑	❑	❑
I felt I couldn't stand it anymore.	❑	❑	❑	❑	❑
I became afraid that the pain would get worse.	❑	❑	❑	❑	❑
I kept thinking of other painful events.	❑	❑	❑	❑	❑
I anxiously wanted the pain to go away.	❑	❑	❑	❑	❑
I couldn't seem to keep it out of my mind.	❑	❑	❑	❑	❑
I kept thinking about how much it hurt.	❑	❑	❑	❑	❑
There was nothing I could do to reduce the intensity of the pain.	❑	❑	❑	❑	❑
I kept thinking about how badly I wanted the pain to stop.	❑	❑	❑	❑	❑
I wondered whether something serious might happen.	❑	❑	❑	❑	❑

**9. In the <u>past 4 months</u>, how often has there been someone…**	**Never**	**Rarely**	**Sometimes**	**Often**	**Always**
To give you information if you need it	❑	❑	❑	❑	❑
To give you helpful advice when dealing with a problem	❑	❑	❑	❑	❑
Who makes you feel appreciated	❑	❑	❑	❑	❑
You can count on to listen to you when you need to talk	❑	❑	❑	❑	❑
With whom to share your most private worries and fears	❑	❑	❑	❑	❑

10. Have any of these events caused you to feel significant stress in the <u>past 4 months</u>? [CHECK ALL THAT APPLY]

- ❑ Change in marital or partner status (death, divorce, separation, marriage)
- ❑ Death of a very close friend or family member
- ❑ Change of work status (business failure, layoff, retirement, return to work)
- ❑ Personal injury or illness
- ❑ Change in health of a very close friend or family member
- ❑ Sexual difficulties
- ❑ Change in financial status
- ❑ Loss or change of caregiver
- ❑ Change in living arrangement
- ❑ Change in ability to engage in physical activities
- ❑ Change in religious and/or social activities
- ❑ Change in sleeping habits
- ❑ Legal problems
- ❑ Difficulty with drugs or alcohol
- ❑ None of the above

11. In the past 4 months, when you visited your doctor, how often did you do the following?
[If you did not visit your doctor in the past 4 months check "Not applicable"]

	Never	Almost never	Sometimes	Fairly often	Very often	Always	Not applicable
Prepare a list of questions for your doctor.	❑	❑	❑	❑	❑	❑	❑
Ask questions about the things you want to know and things you don't understand about your treatment.	❑	❑	❑	❑	❑	❑	❑
Discuss any personal problems that may be related to your illness.	❑	❑	❑	❑	❑	❑	❑

12. Please answer the following questions about your alcohol use in the past 4 months.

**In the past 4 months…**	**Never**	**Monthly or less**	**Two to four times a month**	**Two to three times per week**	**Four or more times a week**
**How often did you have a drink containing alcohol?**	❑	❑	❑	❑	❑

[If "Never" skip to #13]

**In the past 4 months…**	**1 or 2 drinks**	**3 or 4**	**5 or 6**	**7 to 9**	**10 or more**
**How may drinks containing alcohol did you have on a typical day when you were drinking?**	❑	❑	❑	❑	❑

**In the past 4 months…**	**Never**	**Less than monthly**	**Monthly**	**Weekly**	**Daily or almost daily**
**How often did you have six or more drinks on one occasion?**	❑	❑	❑	❑	❑

13. On average, in the past 4 months, how many cigarettes did you smoke a day? _____

**14. In the past 4 months, how many days of the week did you do exercises…**	**0 days**	**1 day**	**2 days**	**3 days**	**4 days**	**5 days**	**6 days**	**7 days**
To get your heart rate up or get aerobic exercise (e.g. brisk walk)	❑	❑	❑	❑	❑	❑	❑	❑
For stretching and flexibility	❑	❑	❑	❑	❑	❑	❑	❑
For strength (e.g. weights, therabands, other strength exercises)	❑	❑	❑	❑	❑	❑	❑	❑
For balance (exercises to improve your balance)	❑	❑	❑	❑	❑	❑	❑	❑

The next two sets of questions ask about the past month.

**15. How much time in the past month…**	**None of the time**	**A little of the time**	**Some of the time**	**A good bit of the time**	**Most of the time**	**All of the time**
Were you discouraged by your health problems?	❑	❑	❑	❑	❑	❑
Were you fearful about your future health?	❑	❑	❑	❑	❑	❑
Was your health a worry in your life?	❑	❑	❑	❑	❑	❑
Were you frustrated by your health problems?	❑	❑	❑	❑	❑	❑

**16. <u>In the past month</u>…**	**Never**	**Rarely**	**Sometimes**	**Usually**	**Always**
I feel alone and apart from others.	❑	❑	❑	❑	❑
I feel left out.	❑	❑	❑	❑	❑
I feel that I am no longer close to anyone.	❑	❑	❑	❑	❑
I feel alone.	❑	❑	❑	❑	❑
I feel lonely.	❑	❑	❑	❑	❑

Thanks so much for answering all of those questions!

The next set of questions ask about your experiences over the <u>past 7 days</u>, so we know what's been going on <u>recently</u>.

**17. During the <u>past 7 days</u>…**	**Without any difficulty**	**With a little difficulty**	**With some difficulty**	**With much difficulty**	**Unable to do**
Are you able to do chores such as vacuuming or yard work?	❑	❑	❑	❑	❑
Are you able to go up and down stairs at a normal pace?	❑	❑	❑	❑	❑
Are you able to go for a walk of at least 15 minutes?	❑	❑	❑	❑	❑
Are you able to run errands and shop?	❑	❑	❑	❑	❑

**18. During the <u>past 7 days</u>…**	**Not at all**	**A little bit**	**Somewhat**	**Quite a bit**	**Very much**
I feel fatigued.	❑	❑	❑	❑	❑
I have trouble <u>starting</u> things because I am tired.	❑	❑	❑	❑	❑
How run-down did you feel on average?	❑	❑	❑	❑	❑
How fatigued were you on average?	❑	❑	❑	❑	❑

**19. In the past 7 days…**	**Very poor**	**Poor**	**Fair**	**Good**	**Very good**
My sleep quality was…	❑	❑	❑	❑	❑

**In the past 7 days…**	**Not at all**	**A little bit**	**Somewhat**	**Quite a bit**	**Very much**
My sleep was refreshing.	❑	❑	❑	❑	❑
I had a problem with my sleep.	❑	❑	❑	❑	❑
I had difficulty falling asleep.	❑	❑	❑	❑	❑

**20. In the past 7 days…**	**Not at all**	**A little bit**	**Somewhat**	**Quite a bit**	**Very much**
How much did pain interfere with your day to day activities?	❑	❑	❑	❑	❑
How much did pain interfere with work around the home?	❑	❑	❑	❑	❑
How much did pain interfere with your ability to participate in social activities?	❑	❑	❑	❑	❑
How much did pain interfere with your household chores?	❑	❑	❑	❑	❑

**21. In the past 7 days…**	**No pain**										**Worst imaginable pain**
How would you rate your pain on **average?**	❑ 0	❑ 1	❑ 2	❑ 3	❑ 4	❑ 5	❑ 6	❑ 7	❑ 8	❑ 9	❑ 10
How would you rate your pain **at its WORST?**	❑ 0	❑ 1	❑ 2	❑ 3	❑ 4	❑ 5	❑ 6	❑ 7	❑ 8	❑ 9	❑ 10

22. In the **past 7 days**…	Didn't have any	A few minutes	Several minutes to an hour	Several hours	A day or two	More than 2 days
When you had pain, how long did it usually last? (If you have several pains, answer in general.)	❑	❑	❑	❑	❑	❑

23. In the **past 7 days**…	Never	Rarely	Sometimes	Usually	Always
I have trouble doing all of my regular leisure activities with others.	❑	❑	❑	❑	❑
I have trouble doing all of the family activities that I want to do.	❑	❑	❑	❑	❑
I have trouble doing all of my usual work (include work at home).	❑	❑	❑	❑	❑
I have trouble doing all of the activities with friends that I want to do.	❑	❑	❑	❑	❑

24. In the **past 7 days**…	Never	Rarely	Sometimes	Often	Always
I felt fearful.	❑	❑	❑	❑	❑
I found it hard to focus on anything other than my anxiety.	❑	❑	❑	❑	❑
My worries overwhelmed me.	❑	❑	❑	❑	❑
I felt uneasy.	❑	❑	❑	❑	❑

25. In the **past 7 days**…	Never	Rarely	Sometimes	Often	Always
I felt worthless.	❑	❑	❑	❑	❑
I felt helpless.	❑	❑	❑	❑	❑
I felt depressed.	❑	❑	❑	❑	❑
I felt hopeless.	❑	❑	❑	❑	❑

26. In the past 7 days…	Never	Rarely	Sometimes	Often	Always
I have been grumpy	❑	❑	❑	❑	❑
I have been feeling like I might snap	❑	❑	❑	❑	❑
Other people have been getting on my nerves	❑	❑	❑	❑	❑
Things have been bothering me more than they normally do	❑	❑	❑	❑	❑
I have been feeling irritable	❑	❑	❑	❑	❑

27. In the past 7 days…	All of the time	Most of the time	More than half of the time	Less than half of the time	Some of the time	At no time
I have felt cheerful and in good spirits.	❑	❑	❑	❑	❑	❑
I have felt calm and relaxed.	❑	❑	❑	❑	❑	❑
I have felt active and vigorous.	❑	❑	❑	❑	❑	❑
I woke up feeling fresh and rested.	❑	❑	❑	❑	❑	❑
My daily life has been filled with things that interest me.	❑	❑	❑	❑	❑	❑

28. Do you have any of the following conditions? For instance, if you have ASTHMA, please put a ✓ in the box next to asthma.

❑ Chronic pain
❑ COPD – Chronic Obstructive Pulmonary Disease
❑ Asthma
❑ Diabetes
❑ High cholesterol
❑ High blood pressure
❑ Ischemic heart disease (coronary artery disease or coronary heart disease)
❑ Atrial Fibrillation
❑ Heart failure
❑ Stroke
❑ BMI 30+ (obesity)
❑ Cancer
❑ Chronic kidney disease
❑ Depression
❑ Osteoporosis
❑ Arthritis
❑ Dizziness/falls/loss of vestibular function

This next set of questions will ask you about everyday things you do in your life, and to what extent you feel limited in doing these activities. Please select the one answer that comes closest to the way you have been feeling.

**29. To what extent do you feel limited in…?**	**Not at all**	**A little**	**Somewhat**	**A lot**	**Completely**
Keeping in touch with others	❑	❑	❑	❑	❑
Visiting friends and family in their homes	❑	❑	❑	❑	❑
Providing care or assistance to others	❑	❑	❑	❑	❑
Working at a volunteer job	❑	❑	❑	❑	❑
Taking part in active recreation	❑	❑	❑	❑	❑
Traveling out of town	❑	❑	❑	❑	❑
Inviting people into your home	❑	❑	❑	❑	❑
Going out with others to public places	❑	❑	❑	❑	❑
Taking part in organized social activities	❑	❑	❑	❑	❑

This next set of questions will ask you about your ability to do specific activities as part of your daily routines. We are interested in your *sense of your ability* to do it on a typical day without the help of someone else, and without the use of a cane, walker, or any other assistive walking device (or a wheelchair or scooter).

It is not important that you actually do the activity on a daily basis. In fact, there may be some activities that you don't do at all. You can still answer these questions by assessing how difficult you think they would be for you to do on an average day.

**30. How much difficulty do you have…** (Remember this is without the help of someone else and without the use of any assistive walking device.)	**None**	**A little**	**Some**	**Quite a lot**	**Cannot do**
Unscrewing the lid off a previously unopened jar without using any devices	❑	❑	❑	❑	❑
Going up & down a flight of stairs inside, using a handrail	❑	❑	❑	❑	❑

	None	A little	Some	Quite a lot	Cannot do
Putting on and taking off long pants (including managing fasteners)	❑	❑	❑	❑	❑
Running ½ mile or more	❑	❑	❑	❑	❑
Using common utensils for preparing meals (e.g., can opener, potato peeler, or sharp knife)	❑	❑	❑	❑	❑
Holding a full glass of water in one hand	❑	❑	❑	❑	❑
Running a short distance, such as to catch a bus	❑	❑	❑	❑	❑
Reaching overhead while standing, as if to pull a light cord	❑	❑	❑	❑	❑
Sitting down in and standing up from a low, soft couch	❑	❑	❑	❑	❑
Putting on and taking off a coat or jacket	❑	❑	❑	❑	❑
Reaching behind your back as if to put a belt through a belt loop	❑	❑	❑	❑	❑
Rip open a package of snack food (e.g. cellophane wrapping on crackers) using only your hands	❑	❑	❑	❑	❑
Pouring from a large pitcher	❑	❑	❑	❑	❑
Getting into and out of a car/taxi (sedan)	❑	❑	❑	❑	❑
Hiking a couple of miles on uneven surfaces, including hills	❑	❑	❑	❑	❑
Going up and down 3 flights of stairs inside, using a handrail	❑	❑	❑	❑	❑
Picking up a kitchen chair and moving it, in order to clean	❑	❑	❑	❑	❑
Using a step stool to reach into a high cabinet	❑	❑	❑	❑	❑
Making a bed, including spreading and tucking in bed sheets	❑	❑	❑	❑	❑
Carrying something in both arms while climbing a flight of stairs (e.g. laundry basket)	❑	❑	❑	❑	❑
Bending over from a standing position to pick up a piece of clothing from the floor	❑	❑	❑	❑	❑
Getting up from the floor (as if you were laying on the ground)	❑	❑	❑	❑	❑
Washing dishes, pots, and utensils by hand while standing at sink	❑	❑	❑	❑	❑
Stepping on and off a bus	❑	❑	❑	❑	❑

Please answer the following questions <u>if you do NOT use a cane, walker, or other walking device</u>.

[If you use a walking device, skip to #32]

**31. How much difficulty do you have…** (Remember this is without the help of someone else and without the use of any assistive walking device.)	**None**	**A little**	**Some**	**Quite a lot**	**Cannot do**
Walking a mile, taking rests as necessary	❑	❑	❑	❑	❑
Going up & down a flight of stairs outside, without using a handrail	❑	❑	❑	❑	❑
Stepping up and down from a curb	❑	❑	❑	❑	❑
Opening a heavy, outside door	❑	❑	❑	❑	❑
Walking around on floor of your home, taking into consideration thresholds, doors, furniture, and a variety of floor coverings	❑	❑	❑	❑	❑
Walking several blocks	❑	❑	❑	❑	❑
Taking a 1 mile, brisk walk without stopping to rest	❑	❑	❑	❑	❑
Walking on a slippery surface, outdoors	❑	❑	❑	❑	❑

Please answer the following questions <u>if you DO use a cane, walker, or other walking device</u>.

[If you do not use a walking device, skip to #33]

**32. When you use your cane, walker, or other walking device, how much difficulty do you have…?**	**None**	**A little**	**Some**	**Quite a lot**	**Cannot do**
Walking a mile, taking rests as necessary	❑	❑	❑	❑	❑
Going up & down a flight of stairs outside, without using a handrail	❑	❑	❑	❑	❑
Stepping up and down from a curb	❑	❑	❑	❑	❑
Opening a heavy, outside door	❑	❑	❑	❑	❑
Walking around on floor of your home, taking into consideration thresholds, doors, furniture, and a variety of floor coverings	❑	❑	❑	❑	❑
Walking several blocks	❑	❑	❑	❑	❑

	None	A little	Some	Quite a lot	Cannot do
Taking a 1 mile, brisk walk without stopping to rest	❑	❑	❑	❑	❑
Walking on a slippery surface, outdoors	❑	❑	❑	❑	❑

This last set of questions are about your experience with ElderTree.
[If you did not have ElderTree for this study, skip the following items.]

EXPERIENCE WITH ELDERTREE

33. Please mark the box below that best describes your experience using ElderTree.

	Not at all	A little bit	Somewhat	Very much so
It helped me learn more about my health issues	❑	❑	❑	❑
It helped my doctor provide better care for me	❑	❑	❑	❑
It helped motivate me to take care of my health	❑	❑	❑	❑
It made me realize I am not alone	❑	❑	❑	❑
It made me happier	❑	❑	❑	❑
I enjoyed the other people on ElderTree	❑	❑	❑	❑
I found it easy to use	❑	❑	❑	❑
It was easy to find what I was looking for	❑	❑	❑	❑
It was glitchy	❑	❑	❑	❑

34. What stopped you or hindered you from using ElderTree or the computer/smart system in general?

____

____

____

____

Please continue to last page

35. If you got a computer or smart system from us, what did you like best about getting the computer or smart system in this study?

____

____

____

____

36. Any other comments or suggestions for us?

____

____

____

____

Thank you for completing this survey.

Recruitment ID#: ______________

Study ID#: ______________

Today's Date: _______________

ElderTree Smart System

8 Month Survey

If you have any questions please contact:

[study coordinator]
[email]
University of Wisconsin – Madison
Center for Health Enhancement Systems Studies
1513 University Avenue
Madison, WI 53706

[tech support and coordinator phones]

Thank you for taking part in this study.

Your answers on this survey are important to us.

Any information you share is confidential.

If you feel uncomfortable with any question, you can skip it.

You are free to stop filling out the survey at any time.

When you are finished with the survey,
please return in the stamped address envelope.

INSTRUCTIONS:
Please check one box per question or row unless otherwise indicated.
Additional instructions are included in brackets [ ].

1. In the <u>past 4 months</u>, how much (if at all) did any of these issues make it hard for you to use a *<u>computer or iPad</u>*? [If you never use a computer or iPad, check "not applicable."]

	**Not at all**	**Slightly**	**Moderately**	**Very**	**Extremely**	**Not applicable**
Vision (even with glasses)	❑	❑	❑	❑	❑	❑
Hearing (even with hearing aids)	❑	❑	❑	❑	❑	❑
Voice Issues (hard to speak clearly)	❑	❑	❑	❑	❑	❑
Lack of knowledge of how to use it	❑	❑	❑	❑	❑	❑
Memory	❑	❑	❑	❑	❑	❑
Other (please specify): ____________________	❑	❑	❑	❑	❑	❑

2. In the <u>past 4 months</u>, how much (if at all) did any of these issues make it hard for you to use a *<u>smart speaker like Alexa, Google Home, or Amazon Echo</u>*? If you never use a smart speaker, check "not applicable."

	**Not at all**	**Slightly**	**Moderately**	**Very**	**Extremely**	**Not applicable**
Vision (even with glasses)	❑	❑	❑	❑	❑	❑
Hearing (even with hearing aids)	❑	❑	❑	❑	❑	❑
Voice Issues (hard to speak clearly)	❑	❑	❑	❑	❑	❑
Lack of knowledge of how to use it	❑	❑	❑	❑	❑	❑
Memory	❑	❑	❑	❑	❑	❑
Other (please specify): ____________________	❑	❑	❑	❑	❑	❑

3. In the past 4 months, how often did you use each the following?

	I don't know what this is/never use	Rarely	Sometimes	Often	Very often
Zoom, Skype, or other video chat	❑	❑	❑	❑	❑
Email	❑	❑	❑	❑	❑
Facebook	❑	❑	❑	❑	❑
Texting	❑	❑	❑	❑	❑
Alexa or similar device	❑	❑	❑	❑	❑

4. In the past 4 months, how many visits have you made to each of the following (Include visits in person, by phone, or video)? [If none, write 0.]

*We know it's hard to remember these details for the past 4 months - do the best you can. We really appreciate it.

_______ Primary care (e.g., primary care doctor, physician assistant, nurse practitioner)

_______ Specialist doctors (e.g., heart, lung, diabetes, cancer, ear/nose/throat, sleep, arthritis, bladder/kidney, women's issues)

_______ Surgeon

_______ Physical therapist, occupational therapist, chiropractor

_______ Counselor, psychologist, psychiatrist, addiction treatment specialist

_______ Ophthalmologist or eye doctor

_______ Dentist

_______ Lab visits (e.g., blood draws, mammogram screening, X-ray, radiation)

_______ Urgent care clinic

_______ Emergency room

5. A key issue for hospitals is if patients are <u>readmitted within 30 days</u> for the same health problem or complications of that problem. Did that happen to you in the <u>past four months</u>?

❑ Yes

❑ No

If Yes, how many times? _______________

6. We would like to know how confident you are in doing certain activities, given your chronic health conditions. Please check the box that corresponds to your confidence that you have been able to do the following tasks regularly in the <u>past 4 months</u>.

**In the <u>past 4 months</u>, how confident have you felt that you could…**	**Not at all confident**	**A little confident**	**Somewhat confident**	**Mostly confident**	**Totally confident**
Keep the fatigue caused by your conditions from interfering with the things you want to do?	❑	❑	❑	❑	❑
Keep the physical discomfort or pain of your conditions from interfering with the things you want to do?	❑	❑	❑	❑	❑
Keep the emotional distress caused by your conditions from interfering with the things you want to do?	❑	❑	❑	❑	❑
Keep any other symptoms or health problems you have from interfering with the things you want to do?	❑	❑	❑	❑	❑
Do the different tasks and activities needed to manage your health conditions so as to reduce your need to see a doctor?	❑	❑	❑	❑	❑
Do things other than just taking medication to reduce how much your conditions affect your everyday life?	❑	❑	❑	❑	❑

**7. In the <u>past 4 months</u>, I tried to manage my health conditions because…**	**Strongly Disagree**	**Disagree**	**Neither agree or disagree**	**Agree**	**Strongly Agree**
Others would be upset with me if I did not	❑	❑	❑	❑	❑
I want to take responsibility for my own health	❑	❑	❑	❑	❑
It is an important choice I really want to make	❑	❑	❑	❑	❑
I feel pressure from others to do so	❑	❑	❑	❑	❑

**8. In the <u>past 4 months</u>, when I was in pain…**	**Not at all**	**A little bit**	**Somewhat**	**Quite a bit**	**Very much**
I worried all the time about whether the pain will end.	❑	❑	❑	❑	❑
I felt I couldn't go on.	❑	❑	❑	❑	❑
It was terrible and I thought it was never going to get any better.	❑	❑	❑	❑	❑
It was awful and I felt that it overwhelmed me.	❑	❑	❑	❑	❑
I felt I couldn't stand it anymore.	❑	❑	❑	❑	❑
I became afraid that the pain would get worse.	❑	❑	❑	❑	❑
I kept thinking of other painful events.	❑	❑	❑	❑	❑
I anxiously wanted the pain to go away.	❑	❑	❑	❑	❑
I couldn't seem to keep it out of my mind.	❑	❑	❑	❑	❑
I kept thinking about how much it hurt.	❑	❑	❑	❑	❑
There was nothing I could do to reduce the intensity of the pain.	❑	❑	❑	❑	❑
I kept thinking about how badly I wanted the pain to stop.	❑	❑	❑	❑	❑
I wondered whether something serious might happen.	❑	❑	❑	❑	❑

**9. In the <u>past 4 months</u>, how often has there been someone…**	**Never**	**Rarely**	**Sometimes**	**Often**	**Always**
To give you information if you need it	❑	❑	❑	❑	❑
To give you helpful advice when dealing with a problem	❑	❑	❑	❑	❑
Who makes you feel appreciated	❑	❑	❑	❑	❑
You can count on to listen to you when you need to talk	❑	❑	❑	❑	❑
With whom to share your most private worries and fears	❑	❑	❑	❑	❑

10. Have any of these events caused you to feel significant stress in the <u>past 4 months</u>? [CHECK ALL THAT APPLY]

- ❑ Change in marital or partner status (death, divorce, separation, marriage)
- ❑ Death of a very close friend or family member
- ❑ Change of work status (business failure, layoff, retirement, return to work)
- ❑ Personal injury or illness
- ❑ Change in health of a very close friend or family member
- ❑ Sexual difficulties
- ❑ Change in financial status
- ❑ Loss or change of caregiver
- ❑ Change in living arrangement
- ❑ Change in ability to engage in physical activities
- ❑ Change in religious and/or social activities
- ❑ Change in sleeping habits
- ❑ Legal problems
- ❑ Difficulty with drugs or alcohol
- ❑ None of the above

11. In the past 4 months, when you visited your doctor, how often did you do the following?
[If you did not visit your doctor in the past 4 months check "Not applicable"]

	Never	Almost never	Sometimes	Fairly often	Very often	Always	Not applicable
Prepare a list of questions for your doctor.	❑	❑	❑	❑	❑	❑	❑
Ask questions about the things you want to know and things you don't understand about your treatment.	❑	❑	❑	❑	❑	❑	❑
Discuss any personal problems that may be related to your illness.	❑	❑	❑	❑	❑	❑	❑

12. Please answer the following questions about your alcohol use in the past 4 months.

**In the past 4 months…**	**Never**	**Monthly or less**	**Two to four times a month**	**Two to three times per week**	**Four or more times a week**
**How often did you have a drink containing alcohol?**	❑	❑	❑	❑	❑

[If "Never" skip to #13]

**In the past 4 months…**	**1 or 2 drinks**	**3 or 4**	**5 or 6**	**7 to 9**	**10 or more**
**How may drinks containing alcohol did you have on a typical day when you were drinking?**	❑	❑	❑	❑	❑

**In the past 4 months…**	**Never**	**Less than monthly**	**Monthly**	**Weekly**	**Daily or almost daily**
**How often did you have six or more drinks on one occasion?**	❑	❑	❑	❑	❑

13. On average, <u>in the past 4</u> months, how many cigarettes did you smoke a day? _____

**14. In the past 4 months, how many days of the week did you do exercises…**	**0 days**	**1 day**	**2 days**	**3 days**	**4 days**	**5 days**	**6 days**	**7 days**
To get your heart rate up or get aerobic exercise (e.g. brisk walk)	❑	❑	❑	❑	❑	❑	❑	❑
For stretching and flexibility	❑	❑	❑	❑	❑	❑	❑	❑
For strength (e.g. weights, therabands, other strength exercises)	❑	❑	❑	❑	❑	❑	❑	❑
For balance (exercises to improve your balance)	❑	❑	❑	❑	❑	❑	❑	❑

For the next question we are asking about falls over the past 6 months (not 4 months).

15. Please answer the follow questions about falls over the past 6 months.

***Note: a fall is when your body goes to the ground without being pushed.**

**In the past 6 months …**	
About how many times have you fallen?	
How many of these falls require medical attention?	

The next two sets of questions ask about the past month.

**16. How much time <u>in the past month</u>…**	**None of the time**	**A little of the time**	**Some of the time**	**A good bit of the time**	**Most of the time**	**All of the time**
Were you discouraged by your health problems?	❑	❑	❑	❑	❑	❑
Were you fearful about your future health?	❑	❑	❑	❑	❑	❑
Was your health a worry in your life?	❑	❑	❑	❑	❑	❑
Were you frustrated by your health problems?	❑	❑	❑	❑	❑	❑

**17. <u>In the past month</u>…**	**Never**	**Rarely**	**Sometimes**	**Usually**	**Always**
I feel alone and apart from others.	❑	❑	❑	❑	❑
I feel left out.	❑	❑	❑	❑	❑
I feel that I am no longer close to anyone.	❑	❑	❑	❑	❑
I feel alone.	❑	❑	❑	❑	❑
I feel lonely.	❑	❑	❑	❑	❑

Thanks so much for answering all of those questions!

The next set of questions ask about your experiences over the <u>past 7 days</u>, so we know what's been going on <u>recently</u>.

**18. During the <u>past 7 days</u>…**	**Without any difficulty**	**With a little difficulty**	**With some difficulty**	**With much difficulty**	**Unable to do**
Are you able to do chores such as vacuuming or yard work?	❑	❑	❑	❑	❑
Are you able to go up and down stairs at a normal pace?	❑	❑	❑	❑	❑
Are you able to go for a walk of at least 15 minutes?	❑	❑	❑	❑	❑
Are you able to run errands and shop?	❑	❑	❑	❑	❑

**19. During the <u>past 7 days</u>…**	**Not at all**	**A little bit**	**Somewhat**	**Quite a bit**	**Very much**
I feel fatigued.	❑	❑	❑	❑	❑
I have trouble <u>starting</u> things because I am tired.	❑	❑	❑	❑	❑
How run-down did you feel on average?	❑	❑	❑	❑	❑
How fatigued were you on average?	❑	❑	❑	❑	❑

**20. In the <u>past 7 days</u>…**	**Very poor**	**Poor**	**Fair**	**Good**	**Very good**
My sleep quality was…	❑	❑	❑	❑	❑

**In the <u>past 7 days</u>…**	**Not at all**	**A little bit**	**Somewhat**	**Quite a bit**	**Very much**
My sleep was refreshing.	❑	❑	❑	❑	❑
I had a problem with my sleep.	❑	❑	❑	❑	❑
I had difficulty falling asleep.	❑	❑	❑	❑	❑

**21. In the <u>past 7 days</u>…**	**Not at all**	**A little bit**	**Somewhat**	**Quite a bit**	**Very much**
How much did pain interfere with your day to day activities?	❑	❑	❑	❑	❑
How much did pain interfere with work around the home?	❑	❑	❑	❑	❑
How much did pain interfere with your ability to participate in social activities?	❑	❑	❑	❑	❑
How much did pain interfere with your household chores?	❑	❑	❑	❑	❑

**22. In the past 7 days…**	**No pain**										**Worst imaginable pain**
How would you rate your pain on **average?**	❑0	❑1	❑2	❑3	❑4	❑5	❑6	❑7	❑8	❑9	❑10
How would you rate your pain **at its WORST?**	❑0	❑1	❑2	❑3	❑4	❑5	❑6	❑7	❑8	❑9	❑10

**23. In the past 7 days…**	**Didn't have any**	**A few minutes**	**Several minutes to an hour**	**Several hours**	**A day or two**	**More than 2 days**
When you had pain, how long did it usually last? (If you have several pains, answer in general.)	❑	❑	❑	❑	❑	❑

**24. In the past 7 days…**	**Never**	**Rarely**	**Sometimes**	**Usually**	**Always**
I have trouble doing all of my regular leisure activities with others.	❑	❑	❑	❑	❑
I have trouble doing all of the family activities that I want to do.	❑	❑	❑	❑	❑
I have trouble doing all of my usual work (include work at home).	❑	❑	❑	❑	❑
I have trouble doing all of the activities with friends that I want to do.	❑	❑	❑	❑	❑

**25. In the past 7 days…**	**Never**	**Rarely**	**Sometimes**	**Often**	**Always**
I felt fearful.	❑	❑	❑	❑	❑
I found it hard to focus on anything other than my anxiety.	❑	❑	❑	❑	❑
My worries overwhelmed me.	❑	❑	❑	❑	❑
I felt uneasy.	❑	❑	❑	❑	❑

**26. In the <u>past 7 days</u>…**	**Never**	**Rarely**	**Sometimes**	**Often**	**Always**
I felt worthless.	❑	❑	❑	❑	❑
I felt helpless.	❑	❑	❑	❑	❑
I felt depressed.	❑	❑	❑	❑	❑
I felt hopeless.	❑	❑	❑	❑	❑

**27. In the <u>past 7 days</u>…**	**Never**	**Rarely**	**Sometimes**	**Often**	**Always**
I have been grumpy	❑	❑	❑	❑	❑
I have been feeling like I might snap	❑	❑	❑	❑	❑
Other people have been getting on my nerves	❑	❑	❑	❑	❑
Things have been bothering me more than they normally do	❑	❑	❑	❑	❑
I have been feeling irritable	❑	❑	❑	❑	❑

**28. In the <u>past 7 days</u>…**	**All of the time**	**Most of the time**	**More than half of the time**	**Less than half of the time**	**Some of the time**	**At no time**
I have felt cheerful and in good spirits.	❑	❑	❑	❑	❑	❑
I have felt calm and relaxed.	❑	❑	❑	❑	❑	❑
I have felt active and vigorous.	❑	❑	❑	❑	❑	❑
I woke up feeling fresh and rested.	❑	❑	❑	❑	❑	❑
My daily life has been filled with things that interest me.	❑	❑	❑	❑	❑	❑

29. Do you have any of the following conditions? For instance, if you have ASTHMA, please put a ✓ in the box next to asthma.

- ❑ Chronic pain
- ❑ COPD – Chronic Obstructive Pulmonary Disease
- ❑ Asthma
- ❑ Diabetes
- ❑ High cholesterol
- ❑ High blood pressure
- ❑ Ischemic heart disease (coronary artery disease or coronary heart disease)
- ❑ Atrial Fibrillation
- ❑ Heart failure
- ❑ Stroke
- ❑ BMI 30+ (obesity)
- ❑ Cancer
- ❑ Chronic kidney disease
- ❑ Depression
- ❑ Osteoporosis
- ❑ Arthritis
- ❑ Dizziness/falls/loss of vestibular function

This next set of questions will ask you about everyday things you do in your life, and to what extent you feel limited in doing these activities. Please select the one answer that comes closest to the way you have been feeling.

**30. To what extent do you feel limited in…?**	**Not at all**	**A little**	**Somewhat**	**A lot**	**Completely**
Keeping in touch with others	❑	❑	❑	❑	❑
Visiting friends and family in their homes	❑	❑	❑	❑	❑
Providing care or assistance to others	❑	❑	❑	❑	❑
Working at a volunteer job	❑	❑	❑	❑	❑
Taking part in active recreation	❑	❑	❑	❑	❑
Traveling out of town	❑	❑	❑	❑	❑
Inviting people into your home	❑	❑	❑	❑	❑
Going out with others to public places	❑	❑	❑	❑	❑
Taking part in organized social activities	❑	❑	❑	❑	❑

This next set of questions will ask you about your ability to do specific activities as part of your daily routines. We are interested in your *sense of your ability* to do it on a typical day <u>without</u> the help of someone else, and <u>without</u> the use of a cane, walker, or any other assistive walking device (or a wheelchair or scooter).

It is not important that you actually do the activity on a daily basis. In fact, there may be some activities that you don't do at all. You can still answer these questions by assessing how difficult you <u>think</u> they would be for you to do on an average day.

**31. How much difficulty do you have…** (Remember this is without the help of someone else and without the use of any assistive walking device.)	**None**	**A little**	**Some**	**Quite a lot**	**Cannot do**
Unscrewing the lid off a previously unopened jar without using any devices	❑	❑	❑	❑	❑
Going up & down a flight of stairs inside, using a handrail	❑	❑	❑	❑	❑
Putting on and taking off long pants (including managing fasteners)	❑	❑	❑	❑	❑
Running ½ mile or more	❑	❑	❑	❑	❑
Using common utensils for preparing meals (e.g., can opener, potato peeler, or sharp knife)	❑	❑	❑	❑	❑
Holding a full glass of water in one hand	❑	❑	❑	❑	❑
Running a short distance, such as to catch a bus	❑	❑	❑	❑	❑
Reaching overhead while standing, as if to pull a light cord	❑	❑	❑	❑	❑
Sitting down in and standing up from a low, soft couch	❑	❑	❑	❑	❑
Putting on and taking off a coat or jacket	❑	❑	❑	❑	❑
Reaching behind your back as if to put a belt through a belt loop	❑	❑	❑	❑	❑
Rip open a package of snack food (e.g. cellophane wrapping on crackers) using only your hands	❑	❑	❑	❑	❑
Pouring from a large pitcher	❑	❑	❑	❑	❑
Getting into and out of a car/taxi (sedan)	❑	❑	❑	❑	❑

	None	A little	Some	Quite a lot	Cannot do
Hiking a couple of miles on uneven surfaces, including hills	❑	❑	❑	❑	❑
Going up and down 3 flights of stairs inside, using a handrail	❑	❑	❑	❑	❑
Picking up a kitchen chair and moving it, in order to clean	❑	❑	❑	❑	❑
Using a step stool to reach into a high cabinet	❑	❑	❑	❑	❑
Making a bed, including spreading and tucking in bed sheets	❑	❑	❑	❑	❑
Carrying something in both arms while climbing a flight of stairs (e.g. laundry basket)	❑	❑	❑	❑	❑
Bending over from a standing position to pick up a piece of clothing from the floor	❑	❑	❑	❑	❑
Getting up from the floor (as if you were laying on the ground)	❑	❑	❑	❑	❑
Washing dishes, pots, and utensils by hand while standing at sink	❑	❑	❑	❑	❑
Stepping on and off a bus	❑	❑	❑	❑	❑

Please answer the following questions <u>if you do NOT use a cane, walker, or other walking device</u>.

[If you use a walking device, skip to #33]

**32. How much difficulty do you have…** (Remember this is without the help of someone else and without the use of any assistive walking device.)	None	A little	Some	Quite a lot	Cannot do
Walking a mile, taking rests as necessary	❑	❑	❑	❑	❑
Going up & down a flight of stairs outside, without using a handrail	❑	❑	❑	❑	❑
Stepping up and down from a curb	❑	❑	❑	❑	❑
Opening a heavy, outside door	❑	❑	❑	❑	❑
Walking around on floor of your home, taking into consideration thresholds, doors, furniture, and a variety of floor coverings	❑	❑	❑	❑	❑
Walking several blocks	❑	❑	❑	❑	❑

	None	A little	Some	Quite a lot	Cannot do
Taking a 1 mile, brisk walk without stopping to rest	❑	❑	❑	❑	❑
Walking on a slippery surface, outdoors	❑	❑	❑	❑	❑

Please answer the following questions <u>if you DO use a cane, walker, or other walking device</u>.

[If you do not use a walking device, skip to #34]

**33. When you use your cane, walker, or other walking device, how much difficulty do you have…?**	None	A little	Some	Quite a lot	Cannot do
Walking a mile, taking rests as necessary	❑	❑	❑	❑	❑
Going up & down a flight of stairs outside, without using a handrail	❑	❑	❑	❑	❑
Stepping up and down from a curb	❑	❑	❑	❑	❑
Opening a heavy, outside door	❑	❑	❑	❑	❑
Walking around on floor of your home, taking into consideration thresholds, doors, furniture, and a variety of floor coverings	❑	❑	❑	❑	❑
Walking several blocks	❑	❑	❑	❑	❑
Taking a 1 mile, brisk walk without stopping to rest	❑	❑	❑	❑	❑
Walking on a slippery surface, outdoors	❑	❑	❑	❑	❑

This last set of questions are about your experience with ElderTree.

[If you did not have ElderTree for this study, skip the following items.]

<u>EXPERIENCE WITH ELDERTREE</u>

34. Please mark the box below that best describes your experience using ElderTree.

	Not at all	A little bit	Somewhat	Very much so
It helped me learn more about my health issues	❑	❑	❑	❑
It helped my doctor provide better care for me	❑	❑	❑	❑

Please continue to last page

	Not at all	A little bit	Somewhat	Very much so
It helped motivate me to take care of my health	❑	❑	❑	❑
It made me realize I am not alone	❑	❑	❑	❑
It made me happier	❑	❑	❑	❑
I enjoyed the other people on ElderTree	❑	❑	❑	❑
I found it easy to use	❑	❑	❑	❑
It was easy to find what I was looking for	❑	❑	❑	❑
It was glitchy	❑	❑	❑	❑

35. What stopped you or hindered you from using ElderTree or the computer/smart system in general?

____

____

____

____

36. If you got a computer or smart system from us, what did you like best about getting the computer or smart system in this study?

____

____

____

____

37. Any other comments or suggestions for us?

____

____

Thank you for completing this survey.
