# Supplementary material for: Using Smart Displays to Implement an eHealth System for Older Adults With Multiple Chronic Conditions: Protocol for a Randomized Controlled Trial
Source: JMIR Res Protoc. 2022 May 5;11(5):e37522. doi: 10.2196/37522 (PMC9121223; doi:10.2196/37522)
Supplement: Multimedia Appendix 2 [file resprot_v11i5e37522_app2.pdf]

# BENEFITS OF PROPOSED RESEARCH

**Project Aim 1.** Develop the smart system platform to deliver the evidence-based Elder Tree system.

**Project Aim 2.** Conduct a randomized clinical trial to test the following hypotheses:

1. Primary outcomes: For patients in the arm receiving Elder Tree (ET) via the smart system platform (ET-SS) versus the arm receiving it on the laptop (ET-LT):
    a. Quality of life will improve more;
    b. Amount of ET use will be higher in month 8.
2. Secondary outcomes: ET-SS (vs. ET-LT) will produce better:
    a. Healthcare use (primary, specialty, urgent, and ER visits; 30-day hospital readmissions);
    b. Clinical outcomes (HbA1c, mg/dl, mmHg, BMI, PHQ-8, and a composite score);
    c. Medication adherence.
3. Mediators:
    a. Use of ET at 4 months will mediate effects of study arms on 8-month ET use;
    b. Negative affect and Self-Determination Theory constructs of coping competence, social relatedness, and intrinsic motivation will mediate the effects of ET use on other outcomes.
4. Moderators: ET-SS (vs. ET-LT) will show greater improvements in primary and secondary outcomes for:
    a. Adults age 65-74 vs. 75+;
    b. Women vs. men;
    c. Patients with 6+ chronic conditions vs. 3-5.

**Benefit 1: Scientific significance in terms of AHRQ's mission and priorities.** This research will directly help achieve five of AHRQ's priorities:

1. An expanded 360$^{o}$ view of healthcare will help policymakers and providers make more informed treatment decisions for high-risk elderly patients with multiple chronic conditions.
    a. The more frequent, more detailed patient data collected in the home, outside the clinic, will expand provider/policymaker views of patient needs (and assets).
    b. Care planning will be based on more reliable data.
    c. More complete data will allow for more sub-analyses.
    d. More expansive data means care planners have more to work with and can dig deeper.
2. Innovation in primary care. ET provides a means to offer care that:
    a. Is home-based, not office-based, a crucial asset during the COVID-19 crisis.
    b. Offers a more frequent picture of how the patient is doing.
    c. Provides care between visits (especially now, when we are told to stay home).
    d. Makes it more likely that people will use it, because it is easy to use.
3. Quality care for low-income, rural, and minority patients. ET provides:
    a. A way to monitor the process and outcomes of care efficiently and conveniently.
    b. Easy access to information and support when office care is inaccessible to patients.
    c. Easy access to information for the clinical team.
4. Using patient-reported outcomes to improve quality and results in primary care. ET will:
    a. Minimize dropout among users, relative to other apps.
    b. Improve adherence (e.g., medication, health tracking).

5. System affordability, efficiency, and quality. ET offers:
    a. Efficient tracking of patient health status and sharing of status information with clinicians.
    b. More frequent status review vs. widely spaced tracking, enabling problems to be caught earlier.
    c. Statistical Process Control to narrow focus to people who need help, when they need it.

**Benefit 2: How this project will advance the field of health IT.** ET will:

1. Minimize dropout. We know people stop using apps in part because:
    a. Entering data can be a hassle. Voice activation will make data entry easier by, e.g., reducing the number of steps required to login and navigate, reducing barriers of physical limitations such as hand tremors and poor vision, etc.
    b. Patients don't perceive enough value relative to data entry burden ("What's in it for me?"). ET's multi-faceted approach addresses a wide range of patient needs, with information, support, self-monitoring, and entertainment services.
2. Enhance ability of the field to develop and use voice activation:
    a. Creating "actions" will be much easier to do in a wider set of applications.
3. Increase monitoring and predicting:
    a. With more continuous, sustained health-tracking data, we can increase the use and validity of key tools such as Natural Language Processing and Machine Learning, for example, to help clinicians understand which patients are most at risk and when they most need intervention.
4. Increase ease of use:
    a. Because of the voice-activated technology, people will find it much easier to add data and use health information.
    b. Many older adults, or those with complex co-morbidities, are tired, stressed, and not ready to learn a lot of new things.
5. Provide a template for other developers:
    a. Systems we develop will be broadly available at no cost.

**Benefit 3: Expected research impact (anticipated or actual) on decision makers.** Because we anticipate less dropout and much more sustained responses to the weekly health-tracking survey, this should help generate:

1. Confidence in research results:
    a. More data and less missingness lead to greater confidence in the data and the conclusions from those data.
2. Speed and efficiency of research:
    a. Having an engaging way to gather data at frequent time intervals potentially increases statistical power substantially, reducing the sample size needed and time for recruitment.
    b. Relatedly, engaging patients via voice activation may reduce attrition, which would also reduce the length of time for recruitment.
3. Large improvements, not small ones:
    a. Many of today's systems produce only barely noticeable improvements. With more data, we are likely to identify opportunities for greater improvement.

4. Strategy for engaging vulnerable patient populations:
    a. The COVID-19 crisis speaks to the need for strategies for delivering 360$^o$ healthcare for seniors and other vulnerable patient populations in ways that don't always involve in-clinic visits.
    b. This study provides data about the effectiveness of a system designed to (1) sustain patients' health monitoring and (2) create a low-burden approach for clinicians to engage in remote monitoring of that information.

## RESPONSE TO CRITIQUES: STRENGTHS AND WEAKNESSES

**CRITIQUE 1:** Addressing a very significant domain using scientifically rigorous methods in an appropriate environment.

**Significance:**

Strengths: Nicely substantiate the gaps in current care and outcomes that their proposed intervention, bolstered by considerable experience with related systems, will address.

Weakness: None noted.

**Investigators:**

Strengths: Excellent experience and expertise.

Weakness: None noted.

**Innovation:**

Strengths: Building on prior platform and focusing on management of MCCs while integrating with primary care workflow.

Weakness: Many apps already exist to help manage chronic conditions. Because most focus on a single condition and those, such as personal health record systems, that could address multiple conditions simultaneously are few in number, **at worst this is a minor weakness.**

*Response: We definitely agree that the challenge isn't a lack of apps but the difficulty of (a) creating an app that can accommodate the complexity of multiple chronic conditions given that there are so many possible combinations of the most common conditions, (b) sustaining use in the population that needs such interventions most, and (c) finding a way to make patients' health-tracking minimally burdensome and maximally useful for clinicians.*

*We note a national survey of US mobile phone users which found that those with no chronic conditions (e.g. hypertension, obesity, diabetes) were more likely to download and use health apps, including apps focused on diet improvement, weight loss, health tracking, and relaxation.[1] Relatedly, qualitative data from patients with multiple chronic conditions suggests that these patients are often reluctant to use health apps for self-tracking because they find it too much work, are struggling with challenges from aging and difficult comorbidities such as depression and chronic pain, and perceive that clinicians don't really care about their health-tracking scores.[2]*

*We have already developed and tested Elder Tree as an intervention that is designed to cope with the complexities of multiple chronic conditions. The array of services on ET are*

*applicable and useful across a wide range of combinations of chronic conditions; thus we provide one consistent, relatively simple approach to support healthcare for patients regardless of which specific combination of conditions they may have. The Clinician Report provides clinicians with a very easy to read overview of changes over time in patients' health tracking.*

*Despite these benefits, the above findings, together with our own observations about decreases in use over time, point to the urgent need to make it easier to engage patients in sustained and more complete use of the intervention. The current project is innovative in working to address these challenges. Moreover, the current COVID-19 crisis makes it clear that there is urgent need to develop and test systems that can engage patients in sustained health tracking and that can help clinicians provide 360 degree care remotely. Understanding the ways new technologies can be leveraged to strengthen telehealth and remote monitoring of vulnerable populations is crucial.*

**Approach:**

Strengths: Project plan is sound. Design, implementation and evaluation methods are detailed and appropriate. Use of extant platform increases likelihood of a successful outcome.

Weakness: No overriding theoretical framework to help tie together the design, implementation, data collection and analysis … absence of such guidance and organization **at worst is a negligible weakness.**

Response*: We acknowledge that there isn't a single theoretical framework that unites all aspects of the project. In part, this reflects the fact that we're addressing several problems simultaneously: how to help patients with multiple chronic conditions, how to sustain their use of the system, and how to communicate effectively with primary care clinicians. Nonetheless, all CHESS systems are driven by Self-Determination Theory (SDT), which asserts that satisfying three psychological needs contributes to adaptive functioning: competence (feeling effective, not overwhelmed), social relatedness (feeling connected to others, not isolated), and intrinsic motivation (feeling autonomous, not coerced).[3] As such, the Elder Tree system itself has a robust theoretical foundation which we test with our mediation analyses.*

*Further, our center developed the NIATx change model, adopted by more than 3,000 health treatment agencies.[4] NIATx led to our research on models to predict and explain implementation success,[5] ingredients of successful change,[6] and diffusion.[7] This research guides the ways in which we work to implement the Elder Tree Clinician Report into clinicians' workflow with minimal burden.*

**Environment:**

Fine.

Weakness: None noted.

**CRITIQUE 2:** A well-designed and thought-out project with a high likelihood of success, interesting health IT.

**Significance:**

Strengths: ET addresses many problems that patients with (multiple) chronic diseases, and especially elderly, have. Current project addresses one problem encountered with their existing tool: continued use and ease of access. If successful, will be a model for others.

<u>Weakness</u>: Unclear how much of ET project could be leveraged by others (API, code?).

> *<u>Response</u>: We apologize that we did not make it clear that we will release our code as open source to serve as a template for other researchers and developers. We will also publish a paper detailing the process of development and rapid cycle testing, as well as any barriers/solutions for development of the voice-activated system. Our goal is to make this project as useful for other developers as possible, given the evident need for such systems.*

**Investigators:**

<u>Strengths</u>: Excellent group with relevant background.

<u>Weakness</u>: None noted.

**Innovation:**

<u>Strengths</u>: Good use of new technology to solve a documented problem.

<u>Weakness</u>: Ease of use may be overestimated (e.g., setting up medication reminders). Can be addressed with someone else doing set-up or by automation (e.g., data from EHR).

> *<u>Response</u>: We completely agree that ease of set-up (and further use) is crucial. As noted earlier, patients with multiple chronic conditions report finding most health tracking apps too much work. That is the primary motivation for the current study, because smart displays and speakers are designed to be relatively easy to use.*
>
> *We have designed Elder Tree to be very easy to both use and set up. Regardless of which study arm they are in, patients start by watching a 5-minute introductory video that explains ET and the technology, then enter information to personalize ET (e.g., their medications and activities) and make a post to the discussion group. A week later, they are encouraged to watch the introductory video again and select another service to learn, thus gradually increasing the services they use. Every 2 months, patients will be prompted to review their ET settings, although they can modify them at any time. These prompts are programmed into the system and will serve the same function even in the "real world" outside the confines of the study. Also, because it's easy for users to forget specific functionality, we have developed and built in short explanatory videos about all ET functions that are easy to access from any part of the system.*

**Approach:**

<u>Strengths</u>: Good strategy and well thought out study.

<u>Weakness</u>: Some selection bias in recruitment – those who like new gadgets may be more likely to participate, inflating effect sizes.

> *<u>Response</u>: We have developed a number of strategies to minimize this issue. Patients are recruited by a letter sent from their primary care clinic, describing a study designed to help older adults with chronic conditions. The specific technologies being offered are not explicitly mentioned. Patients give consent before being randomized to condition, and we will carefully track all dropouts and reasons for dropouts consistent with CONSORT standards.*

<u>Weakness</u>: Training bias may still exist though this seems addressed with having "a friendly graduate student" available for participants to help and setup.

*<u>Response</u>: In fact, virtually all of our training is provided by people who are not "techies." The trainers are non-threatening, patient, and sensitive people who take the time needed to train and do so in a respectful manner.*

<u>Minor weakness</u>: A minor weakness to consider is some type of backup for when the wifi falls out. What would happen to medication reminders? How would users be notified that it's a wifi problem and not something the user did wrong (as many elderly may assume).

*Response: This is an excellent point. First, we will make all participants aware that no system of reminders is infallible, that wifi and system failures do occur and that they should keep whatever systems they currently use as a backup. Within the confines of the study, we offer excellent technical support so that participants can call with questions. For patients who report frequent wifi failures, we can provide a hotspot so that they have a two-tier system.*

*Additionally, understanding the impact of such challenges as loss of wifi on the effectiveness of an eHealth intervention is valuable in itself. Our goal is that the project will help identify such real-world barriers to system use, as well as strategies for surmounting them.*

**Environment:**

Excellent team.

<u>Weakness</u>: None noted.

**CRITIQUE 3:** If successful, will provide a smart display/speaker-enabled solution that is easier to use to make ET more accessible and more effective for older adults, which may in turn lead to improvements in their health and quality of life.

**Significance**:

<u>Strengths</u>: Adoption of smart displays/speakers has risen significantly. They may be perfect for delivering eHealth interventions to older adults but no large RCTs have been conducted.

<u>Weakness</u>: None noted.

**Investigators:**

<u>Strengths</u>: PI is renowned scholar, multi-disciplinary team, addition of Dr. Mutlu who is an expert in human-centered design of robotic technologies.

<u>Weakness</u>: None noted.

**Innovation:**

<u>Strengths</u>: Use of smart displays and smart speakers to deliver health interventions to the elderly population is innovative.

<u>Weakness</u>**:** None noted.

**Approach:**

<u>Strengths</u>: Robust trial design, careful cost-benefit consideration.

Weakness: Remains uncertain if complex information systems can be delivered via smart displays/speakers. Would be good if there were more thorough pilot study and working prototype with demonstrated usability.

*Response: Because this is a highly innovative project, there is minimal prior research on the use of voice-activated systems to help older adults with multiple chronic conditions. We have had 11 older adults pilot-test a voice-navigated speaker (the Google Mini) at home for 5 weeks. During that time, we asked them to try something new (e.g., make a phone call, set a reminder). All 11 felt the speaker was "very easy to use," 10 "strongly agreed" that it would improve quality of life for older adults, and all would recommend it to others. We have gone back to that panel of older adults to test a voice-activated screen during the past 6 months; their input has informed our team's development of voice-activated tools needed for ET.*

*We do recognize that these data are relatively limited. However, we are confident that we have the expertise and capacity to design the voice-activated system. Dr. Mutlu's lab regularly builds such applications where voice assistants, virtual assistants, computers, or robots hold conversational interactions with people. He also teaches a class, "Building User Interfaces," wherein he teaches students how to build task-oriented, voice-based interfaces. This year, they built an e-commerce clothing site on which customers made purchases using a voice assistant. Based on this expertise, we have developed a clear set of procedures for development and rapid-cycle testing.*

**Environment**:

Excellent.

Weakness: None noted.

## REFERENCES


1. Robbins R, Krebs P, Jagannathan R, Jean-Louis G, Duncan DT. Health app use among US mobile phone users: analysis of trends by chronic disease status. JMIR mHealth and uHealth. 2017;5(12):e197.
2. Ancker JS, Witteman HO, Hafeez B, Provencher T, Van de Graaf M, Wei E. "You get reminded you're a sick person": personal data tracking and patients with multiple chronic conditions. Journal of Medical Internet Research. 2015;17(8):e202.
3. Ryan RM, Deci EL. Self-determination theory and the facilitation of intrinsic motivation, social development, and well-being. American Psychologist. 2000;55(1):68-78.
4. McCarty D, Gustafson DH, Wisdom JP, Ford J, Choi D, Molfenter T, Capoccia V, Cotter F. The Network for the Improvement of Addiction Treatment (NIATx): enhancing access and retention. Drug and Alcohol Dependence. 2007;88(2-3):138-45. PMCID: PMC1896099.
5. Gustafson DH, Brennan PF, Hawkins RP. Investing in e-health: what it takes to sustain consumer health informatics. In: Hannah KJ, Ball MJ, editors. Health Informatics. New York: Springer Science+Business Media, LLC; 2007.
6. Gustafson DH, Hundt AS. Findings of innovation research applied to quality management principles for health care. Health Care Management Review. 1995;20(2):16-33.
7. Hoffman KA, Ford JH, Choi D, Gustafson DH, McCarty D. Replication and sustainability of improved access and retention within the Network for the Improvement of Addiction Treatment. Drug and Alcohol Dependence. 2008;98.
